# Multifaceted activation of STING axis upon Nipah and measles virus-induced syncytia formation

**DOI:** 10.1371/journal.ppat.1012569

**Published:** 2024-09-16

**Authors:** Lucia Amurri, Claire Dumont, Rodolphe Pelissier, Olivier Reynard, Cyrille Mathieu, Julia Spanier, Bernadett Pályi, Daniel Déri, Ludovic Karkowski, Claudia Gonzalez, Jennifer Skerra, Zoltán Kis, Ulrich Kalinke, Branka Horvat, Mathieu Iampietro

**Affiliations:** 1 CIRI, Centre International de Recherche en Infectiologie, INSERM U1111, CNRS, UMR5308, Univ Lyon, Université Claude Bernard Lyon, École Normale Supérieure de Lyon, Lyon, France; 2 Institute for Experimental Infection Research, TWINCORE, Centre for Experimental and Clinical Infection research, Hanover, Germany; 3 National Biosafety Laboratory, National Center for Public Health and Pharmacy, Budapest, Hungary; 4 Semmelweis University, Institute of Medical Microbiology, Budapest, Hungary; 5 European Research Infrastructure on Highly Pathogenic Agents (ERINHA-AISBL), Brussels, Belgium; Division of Clinical Research, UNITED STATES OF AMERICA

## Abstract

Activation of the DNA-sensing STING axis by RNA viruses plays a role in antiviral response through mechanisms that remain poorly understood. Here, we show that the STING pathway regulates Nipah virus (NiV) replication *in vivo* in mice. Moreover, we demonstrate that following both NiV and measles virus (MeV) infection, IFNγ-inducible protein 16 (IFI16), an alternative DNA sensor in addition to cGAS, induces the activation of STING, leading to the phosphorylation of NF-κB p65 and the production of IFNβ and interleukin 6. Finally, we found that paramyxovirus-induced syncytia formation is responsible for loss of mitochondrial membrane potential and leakage of mitochondrial DNA in the cytoplasm, the latter of which is further detected by both cGAS and IFI16. These results contribute to improve our understanding about NiV and MeV immunopathogenesis and provide potential paths for alternative therapeutic strategies.

## Author summary

Viruses belonging to *Paramyxoviridae* family, such as Nipah and measles virus, represent a threat for public health due to recurring zoonotic spillover events or increasing epidemic episodes, respectively. In our previous work, we demonstrated the involvement of the DNA-sensing cGAS/STING axis of innate immunity in the control of Nipah and measles virus infections. However, the cellular and molecular mechanisms of STING activation by these RNA viruses remained obscure. Here, we show first that STING regulates Nipah virus infection *in vivo* and is activated both canonically and non-canonically by cGAS and IFI16 DNA sensors; respectively, during *Paramyxovirus* infections. Moreover, we describe that syncytia formation caused by both Nipah and measles viruses perpetrate mitochondrial perturbation, thus responsible for the leakage of DNA. Globally, we linked the events demonstrating that viral-induced syncytia formation triggers the leakage of mitochondrial DNA in the cytoplasm and its further sensing by cGAS and IFI16.

## Introduction

Innate immunity plays a critical role in antiviral responses, and cyclic guanosine monophosphate-adenosine monophosphate (cGAMP) synthase/stimulator of IFN genes (cGAS/STING) is the main innate immune signaling axis involved in the recognition of cytoplasmic double-strand DNA (dsDNA) [1]. The STING pathway can be activated by both non-self-DNA of invading pathogens and self-DNA derived from damaged organelles in cancerous, senescent or infected cells [2–4]. In the presence of immunostimulatory DNA, the sensor cGAS binds the minor groove of dsDNA in a sequence-independent manner through its zinc-ion-binding domain, thus oligomerizing and catalyzing the synthesis of cyclic dinucleotide cGAMP [5,6]. Then, cGAMP acts as a second messenger by binding and activating STING, inducing its oligomerization and translocation from the endoplasmic reticulum (ER) to the Golgi apparatus and the perinuclear puncta [7]. There, STING is phosphorylated by TANK-binding kinase 1 (TBK1), resulting in the recruitment of interferon (IFN) regulatory factor 3 (IRF3) and the induction of type I IFNs (IFN-I) and other cytokines [8–12]. While being identified as a major actor in the sensing of exogenous pathogenic DNA, the STING axis also plays an important role in the response against various enveloped RNA viruses, such as flaviviruses, coronaviruses and orthomyxoviruses, through indirect mechanisms, as previously described [13,14]. In addition, we have recently demonstrated the involvement of the STING axis in the control of Nipah (NiV) and measles virus (MeV) infection [15,16], although the mechanism of virus-induced activation of STING axis remained unclear.

NiV and MeV are both *Mononegavirales* belonging to the *Paramyxoviridae* family, which is characterized by an enveloped virion containing a non-segmented single-stranded RNA (ssRNA) genome of negative polarity [17,18]. NiV is an emerging highly pathogenic virus mainly transmitted through the respiratory or oral route by *Pteropus* fruit bats, which are widespread in South-East Asia, Australia and Africa and constitute an asymptomatic host reservoir [19,20]. The symptoms of Nipah disease include severe pneumonia and encephalitis with up to 100% lethality and no vaccine or therapeutic against NiV are currently available. In addition, several factors, such as intensive farming and global warming, can contribute to an uncontrolled spreading of this virus with pandemic potential [21]. Indeed, NiV is classified among chemical, radiological, biological and nuclear threats and belongs to the World Health Organization Blueprint list of priority pathogens for research and development, underlying the urge to improve our understandings on NiV immunopathogenesis to develop innovative therapeutic strategies.

MeV is a highly contagious reemerging virus transmitted through the respiratory route. Measles disease symptoms are ranging from mild (fever, cough, skin rash and conjunctivitis) to severe (pneumonia and encephalitis) [22]. Moreover, due to its ability to infect immune cells, MeV induces a transient immune suppression known as "immune amnesia”, thus increasing the risk of opportunistic infections [23,24]. Despite the availability of a safe and effective vaccine, measles outbreaks are resurging in recent years due to a suboptimal vaccination coverage, which has been worsened by the delay in routine children vaccinations during COVID-19 pandemic [25,26]. While NiV glycoprotein (G) binds cellular ephrin B2 and B3 receptors, which are ubiquitously expressed and highly conserved in mammalians [27], MeV hemagglutinin (H) interacts with CD150 and Nectin-4 receptors expressed on immune and epithelial cells, respectively [28–30]. In addition, MeV H from vaccinal strains also interacts with the ubiquitously expressed CD46 receptor [31,32]. The expression of G/H and fusion (F) envelope glycoproteins on the surface of infected cells during viral replication induces cell-to-cell fusion, leading to the formation of multinucleated giant cells named syncytia both *in vitro* and *in vivo* [33,34].

We have recently demonstrated that *Paramyxovirus* infection activates STING axis both *in vitro* and *in vivo* along canonical RNA sensors such as Toll-like receptors (TLR) and RIG-I-like receptors (RLR) [15,16]. We observed a synergistic and non-redundant role of STING together with mitochondrial antiviral signaling protein (MAVS) and myeloid differentiation primary response 88 (MyD88) in the control of NiV infection in mice [16]. Moreover, it has been recently observed that virus-induced downregulation of mitochondrial biogenesis triggers the release of mitochondrial DNA (mtDNA) in the cytoplasm and activates cGAS during MeV infection [35]. STING can undergo non-degradative K63-linked ubiquitination by multiple E3 ubiquitin ligases, such as tripartite motif containing protein 32 (TRIM32), TRIM56 or tumor necrosis factor receptor-associated factor 6 (TRAF6), resulting in the induction of nuclear factor kappa B (NF-κB) and subsequent expression of inflammatory cytokines [36–38]. In addition to cGAS, another DNA sensor, IFNγ inducible protein 16 (IFI16), has been described as an alternative activator of STING [39,40]. IFI16 binds damaged DNA through its hematopoietic expression-IFN inducible-nuclear localization (HIN) domain and activates STING through a cGAMP-independent mechanism in complex with tumor suppressor p53 and TRAF6 [40,41]. While cGAMP predominantly induces STING phosphorylation and triggers IFN-I expression through TBK1 and IRF3, IFI16-dependent STING activation is associated to a prevalence of poly-ubiquitinated STING and NF-κB activation [40,42]. Thus, deciphering the function of the different stimulators of STING is fundamental to understand the impact on STING axis activation and further gene expression.

Here, we report that, concomitantly to our previous observations [16], the deficiency of STING (knock out, KO) affects the control of NiV infection in mice *in vivo*. Indeed, our animal experiment revealed an increase in viral replication that is associated with a decrease in CXC motif chemokine ligand 10 (CXCL10) expression and impaired production of neutralizing antibodies in KO animals compared to the wild-type (WT). We have then investigated the cellular and molecular profiles associated with STING activation during *Paramyxovirus* infection *in vitro* and showed that STING is activated in parallel through two distinct sensors. While the canonical cGAS/STING pathway is mainly involved in the implementation of the IFN-I response, the non-canonical axis involving IFI16 has a prevalent effect on STING-dependent activation of NF-κB subunit p65, mainly responsible for the expression of inflammatory cytokines. Moreover, our results revealed that STING activation is dependent on virus-induced membrane fusion following both NiV and MeV infection. Finally, we demonstrate that syncytia formation triggers mitochondrial loss of membrane potential in infected cells and the release of mtDNA in the cytoplasm, which is further sensed by both cGAS and IFI16. Overall, our study highlights novel aspects of STING axis activation during RNA virus infections and provides key elements to better understand immunovirological mechanisms involved during *Paramyxovirus* infection.

## Results

### STING plays a role in the control of NiV infection in mice

**Fig 1:**
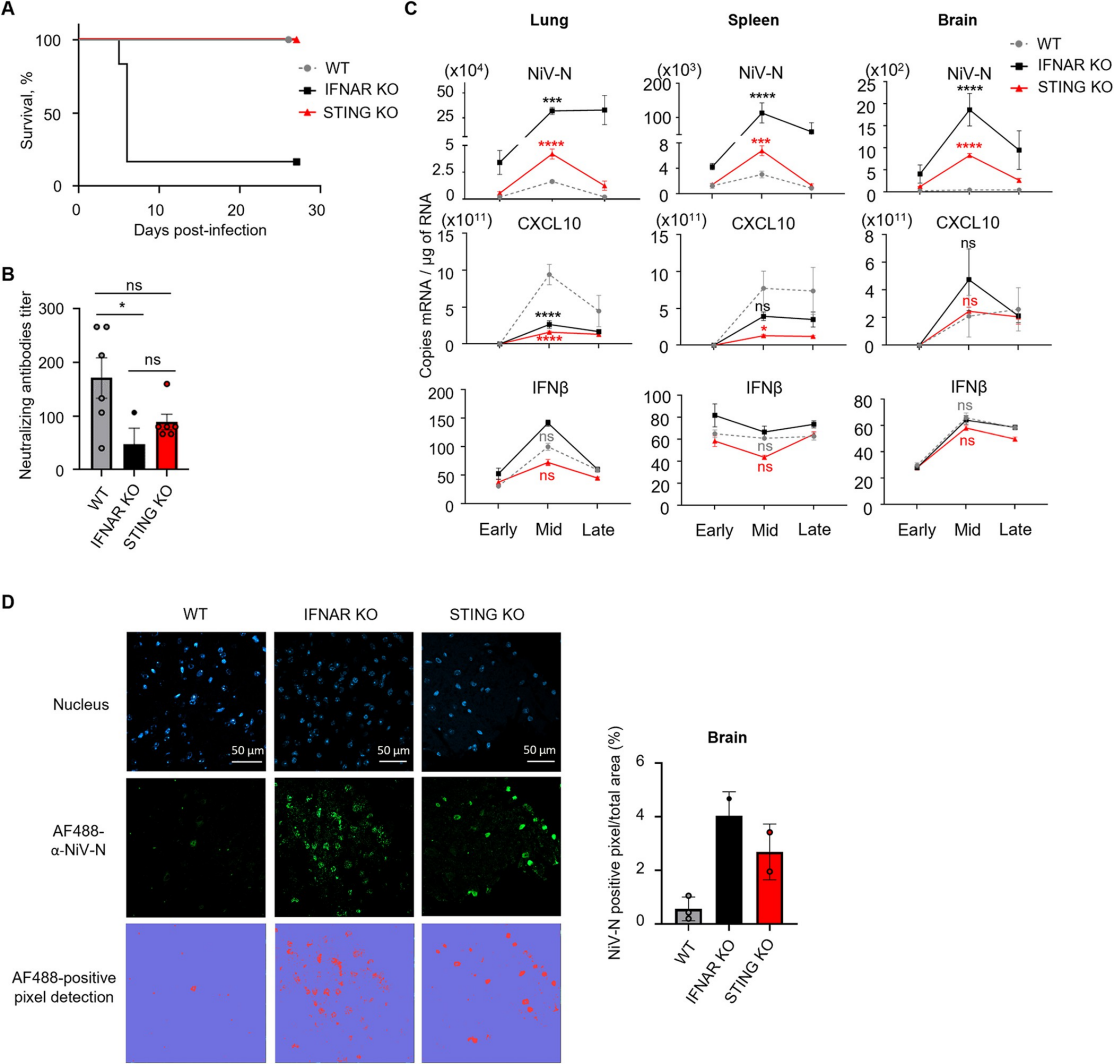
STING plays a role in the control of NiV infection in mice. **(A)** Wild-type (WT), IFNα/β receptor (IFNAR) KO and STING KO C57BL/6 mice were infected intraperitoneally with 10^6^ plaque-forming unit (PFU) of NiV-Malaysia (NiV) and observed during 28 days post-infection. Survival of NiV-infected mice was followed up for 28 days (n = 5 WT mice, n = 6 IFNAR KO mice and n = 5 STING KO mice). For kinetics analysis, results were grouped into early (from day 0+4h to day 2 post infection), mid (from day 2 to day 7) and late (from day 7 to day 28) phase of infection (3 to 6 animals per group). **(B)** α-NiV neutralizing antibodies titer measured in serum of 6 WT mice euthanized at day 28, 3 IFNAR KO mice euthanized at day 7 or day 27 and 6 STING KO mice euthanized at day 28. **(C)** NiV nucleoprotein (NiV-N), CXCL10 and IFNβ mRNA levels in murine lungs, spleens and brains harvested at early, mid or late phase were assessed by RT-qPCR. Data are represented as mean ± standard error of the mean (SEM). The difference between IFNAR KO and WT (in grey) or STING KO and WT (in red) was analyzed using two-way analysis of variance, followed by Tukey multiple comparison test: ns (not significant); *p<0.05; ***p<0.001; ****p<0.0001 compared to WT condition. **(D)** Brains of WT, IFNAR KO and STING KO C57BL/6 mice were harvested at day 7 post NiV infection and analyzed by fluorescence microscopy after staining with DAPI and anti-NiV-N antibody. The percentage of NiV-N positive pixels/total area was quantified with QuPath-0.4.3.

While our previous study demonstrated a synergistic role of STING in addition to RNA-sensing axes of innate immunity, namely TLR/MyD88 and RLR/MAVS pathways [16], the impact of STING alone on NiV infection *in vivo* remained unknown. Therefore, we compared the infection with NiV-Malaysia in wild-type (WT), IFNα/β receptor (IFNAR) KO and STING KO mice for 28 days (Fig 1). While all WT mice survived NiV infection without developing disease, as previously described [43], 5 out of 6 IFNAR KO mice died following infection. Similarly to WT mice, all STING KO mice survived the NiV challenge without developing clinical symptoms (Fig 1A). However, despite their survival, the neutralizing antibodies titer in the serum of STING KO mice was equivalent to IFNAR KO mice that reached endpoint euthanasia and reduced compared to WT (Fig 1B). Then, to further characterize the impact of STING in the control of NiV infection, we harvested lung, spleen and brain of mice at early (day 0-day 2), mid (day 2-day 7), or late (day 7-day 28) phase of infection and analyzed viral replication and cytokine expression by RT-qPCR (Fig 1C). CXCL10 is known to be an important chemo-attractant involved in the generation of inflammatory immune response and overexpressed during NiV infection [44]. Although the lack of STING did not affect animal survival and despite similar levels of IFNβ mRNA, STING KO mice displayed significantly higher viral NiV-N mRNA levels in all tested organs, followed by lower expression of CXCL10 in the brain and lung in the mid phase of infection compared to WT mice, as observed in IFNAR KO control mice (Fig 1C). These observations were confirmed at proteic level, as we detected more NiV N antigen in brains harvested from STING KO mice compared to WT mice at day 7, thus representing the peak of infection (Fig 1D. Overall, these results demonstrate an important role of STING axis in the control of NiV infection *in vivo* thus confirming the involvement of this DNA sensor in parallel to RNA-sensing pathways.

### STING pathway controls both IFN-I and NF-κB p65 responses following NiV infection

**Fig 2:**
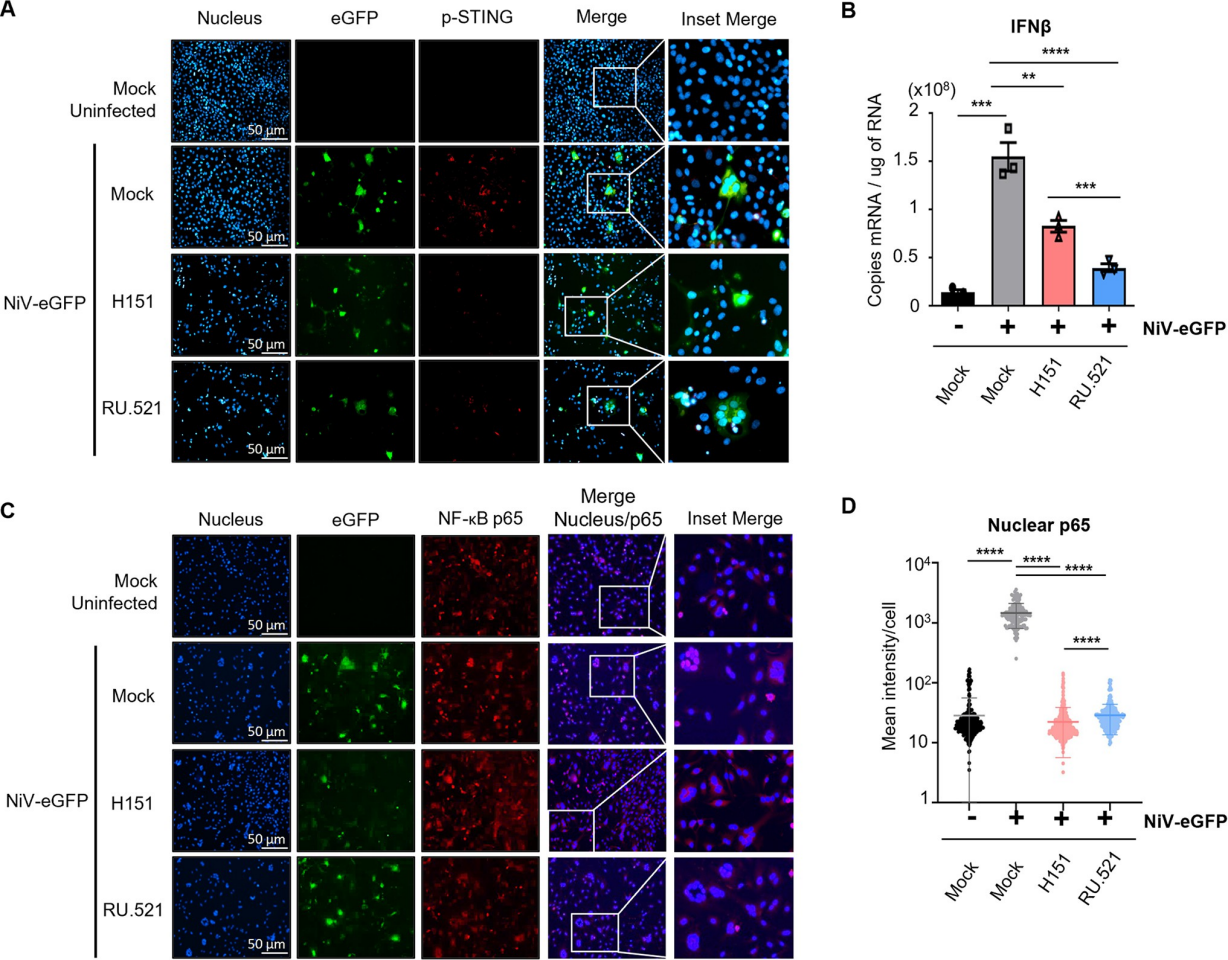
STING pathway controls IFNβ and NF-κB p65 responses following NiV infection. Human pulmonary microvascular endothelial cells (HPMEC) were infected with NiV-eGFP at a MOI of 1 for 48h and treated or non-treated with a STING inhibitor (H151) or a cGAS inhibitor (RU.521) at 10 μM or 10 μg/ml, respectively, 1h before infection. (A, C) Cells were analyzed by fluorescence microscopy following fixation and immunofluorescence staining of phospho-STING (p-STING) and NF-κB p65. (B) Cells were harvested and analyzed by RT-qPCR for IFNβ expression. Data from 3 independent experiments are represented as mean ± SEM. All samples were analyzed using Kruskal-Wallis and Conover post-hoc test, *p<0.05; **p<0.01; ****p<0.0001 compared to NiV-infected WT condition. (D) The mean intensity of nuclear p65 fraction/cell was calculated using QuPath-0.4.3 on at least 130 events per condition. For infected conditions, only infected cells were analyzed. Data are represented as mean ± SD. All samples were analyzed using Kruskal Wallis and Dunn’s multiple comparison test, ****p<0.0001 compared to NiV-infected WT condition.

To investigate how STING pathway regulates the innate immune response to NiV infection in human main cellular targets, we analyzed modulation of IFN-I through IFNβ expression and NF-κB p65 activation in human pulmonary microvascular endothelial cells (HPMEC) infected with recombinant NiV-eGFP and treated or not with a specific STING inhibitor (H-151) or cGAS inhibitor (RU.521) (Fig 2). NiV infection induced the activation of STING through phosphorylation, as observed by fluorescent microscopy, while H151 and RU.521 treatments strongly reduced phospho-STING (p-STING) staining at similar levels (Fig 2A). Also, we determined that NiV infection was associated with a significant increase in IFNβ expression, which was decreased by both H151 and RU.521 treatments, demonstrating a STING-dependent IFNβ induction in response to NiV (Fig 2B).

In parallel, we evaluated activation of NF-κB p65 subunit and observed its nuclear translocation following NiV infection, that was significantly reduced by both H-151 and RU.521 treatments (Fig 2C and 2D). Interestingly, cGAS specific inhibition through RU.521 resulted in a significantly reduced inhibition of p65 nuclear translocation, compared to STING inhibition by H-151 (Fig 2D). Altogether, these data indicate that STING pathway contributes to trigger both IFN-I and NF-κB p65 responses following NiV infection. Moreover, our data suggested that a cGAS-independent stimulus could be also involved in the activation of NF-κB pathway, thus implying the participation of an alternative DNA sensor during NiV infection.

### STING-associated sensors cGAS and IFI16 are involved in the control of NiV and MeV infection

**Fig 3:**
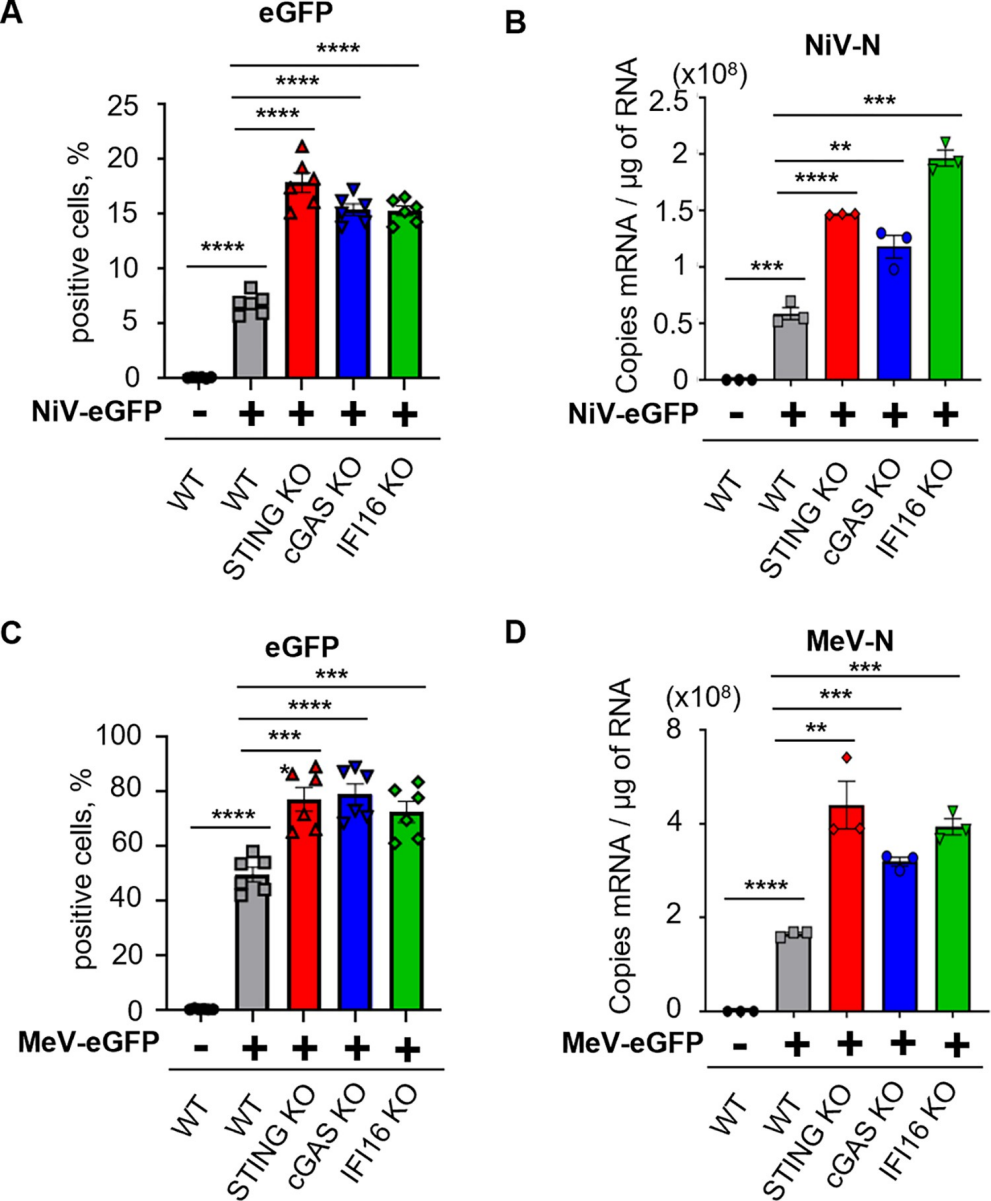
STING-associated sensors cGAS and IFI16 are involved in the control of NiV and MeV infection. WT, STING KO, cGAS KO or IFI16 KO THP-1 cells were infected with NiV-eGFP at a MOI of 0.3 or MeV-eGFP at a MOI of 0.1 for 48h. (A, C) eGFP expression was evaluated by flow cytometry in NiV-eGFP (A) or MeV-eGFP (C) infected cells (n = 6). Data are represented as mean ± SEM. All samples were analyzed using one-way analysis of variance, followed by Tukey’s multiple comparison test, ***p<0.001; ****p<0.0001 compared to NiV- or MeV-infected WT condition. (B, D) Cells were harvested and analyzed by RT-qPCR for NiV-N (C) or MeV-N (F) expression (n = 3). Data are represented as mean ± SEM. All samples were analyzed using one-way analysis of variance, followed by Tukey’s multiple comparison test, **p<0.01; ***p<0.001; ****p<0.0001 compared to NiV- or MeV-infected WT condition.

To further analyze a possible role of an alternative STING-activating DNA sensor during NiV and MeV infection, we investigated the potential contribution of IFI16 as a DNA sensor in parallel to cGAS. Indeed, we previously demonstrated that STING undergoes non-degradative K63-linked ubiquitination following NiV and MeV infection [16] and IFI16 was shown to predominantly trigger STING ubiquitination [40]. To address the role of each factor in the innate response against viral infection, we infected susceptible monocytic THP-1 cells WT or KO for STING, cGAS or IFI16 with NiV-eGFP (Fig 3A and 3B) or MeV-eGFP (Fig 3C and 3D). Our data demonstrated an increased viral propagation in cell cultures lacking each analyzed molecule involved in the sensing of DNA, compared to WT cells (Figs 3A, 3C, S1A and S1B). Moreover, our flow cytometry data were further confirmed by RT-qPCR, demonstrating that NiV-N and MeV-N RNA levels were significantly increased in STING KO, cGAS KO and IFI16 KO cells compared to WT THP-1 cells (Fig 3B and 3D). Overall, these results determined the involvement of IFI16 along cGAS in the control of NiV and MeV infections by STING axis.

### cGAS promotes phosphorylation of STING and IFI16 favors NF-κB p65 activation following NiV and MeV infection

**Fig 4:**
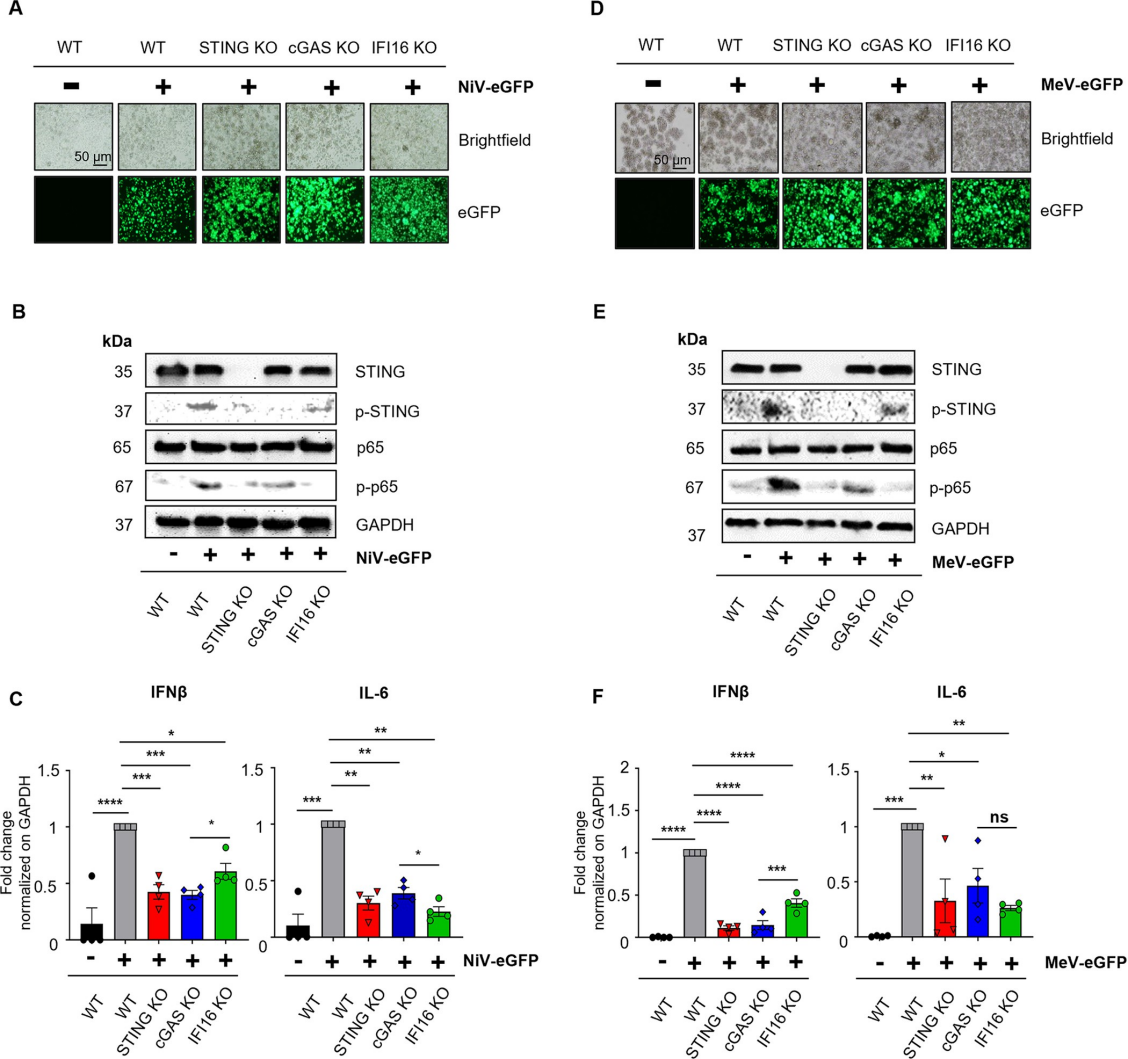
cGAS promotes phosphorylation of STING and IFI16 favors NF-κB p65 activation following NiV and MeV infection. WT, STING KO, cGAS KO or IFI16 KO THP-1 cells were infected with NiV-eGFP (A-C) or MeV-eGFP (D-F) at a MOI of 1 for 48h. (A, D) Representative image of eGFP expression from 3 independent experiments, evaluated by fluorescence microscopy in NiV- (A) and MeV- (D) infected cells. (B, E) NiV- (B) and MeV- (E) infected cells were analyzed for NF-κB p65, phospho-p65 (p-p65), STING, phospho-STING (p-STING) and GAPDH expression by western blot analysis. (C, F) NiV- (C) or MeV- (F) infected cells were harvested and analyzed by RT-qPCR for IFNβ and IL-6 expression (n = 4). Data are represented as mean ± SEM. All samples were analyzed using one-way analysis of variance, followed by Tukey’s multiple comparison test, *p<0.05; **p<0.01; ***p<0.001; ****p<0.0001.

To investigate whether cGAS and IFI16 could differently activate STING and its subsequent pathway, we analyzed protein levels of p-STING and phospho-p65 NF-κB (p-p65), along with IFNβ and IL-6 mRNA levels in WT, STING KO, cGAS KO and IFI16 KO THP-1 cells infected with either NiV-eGFP (Fig 4A–4C) or MeV-eGFP (Fig 4D–4F). These targets were chosen in an attempt to dissect two axes of STING activation: one canonical axis mainly involving p-STING/IFNβ and the other non-canonical axis mostly represented by p-p65/IL-6. First, to maximize virus-induced signaling activation we verified that all our cultures were efficiently infected by following eGFP immunofluorescence (Fig 4A and 4D). Western blot analyses then demonstrated that p-STING and p-p65 levels were strongly increased in NiV- and MeV-infected WT cells compared to uninfected cultures (Fig 4B and 4E). In addition, our data confirmed that STING is involved in the activation of NF-κB p65 following NiV and MeV infection as STING KO cultures displayed reduced levels of p-p65 compared to WT cells (Fig 4B and 4E). Interestingly, cGAS KO and IFI16 KO presented opposite effects on STING and NF-kB p65 activation following both NiV and MeV infection. Though the absence of cGAS had a strong impact in reducing p-STING levels while p-p65 remained high, the lack of IFI16 generated an important diminution of p-p65 levels while maintaining a relatively high amount of p-STING (Fig 4B and 4E). These observations were corroborated also at cytokine level, as both IFNβ and IL-6 mRNA amounts were increased in WT THP-1 cultures following NiV and MeV infection (Fig 4C and 4F). Moreover, both cytokines’ expression was significantly reduced in the absence of STING, confirming its global action in cytokine response to paramyxoviruses. Finally, while cGAS KO THP-1 cells displayed an important reduction in IFNβ mRNA levels and relatively maintained IL-6 amounts, IFI16 KO THP-1 cells exerted opposite effects with relative decrease of IFNβ mRNA quantity and a major reduction in IL-6 mRNA levels (Fig 4C and 4F). Altogether, these data confirm the presence of two axes involved in the activation of STING pathway following *Paramyxovirus* infections, where cGAS mostly favors STING phosphorylation and IFN-I response, while IFI16 mainly triggers NF-κB activation and pro-inflammatory cytokines response possibly through STING ubiquitination as previously described [16,40].

### Virus-induced syncytia lead to STING activation and expression of IFN-I and inflammatory cytokines

**Fig 5:**
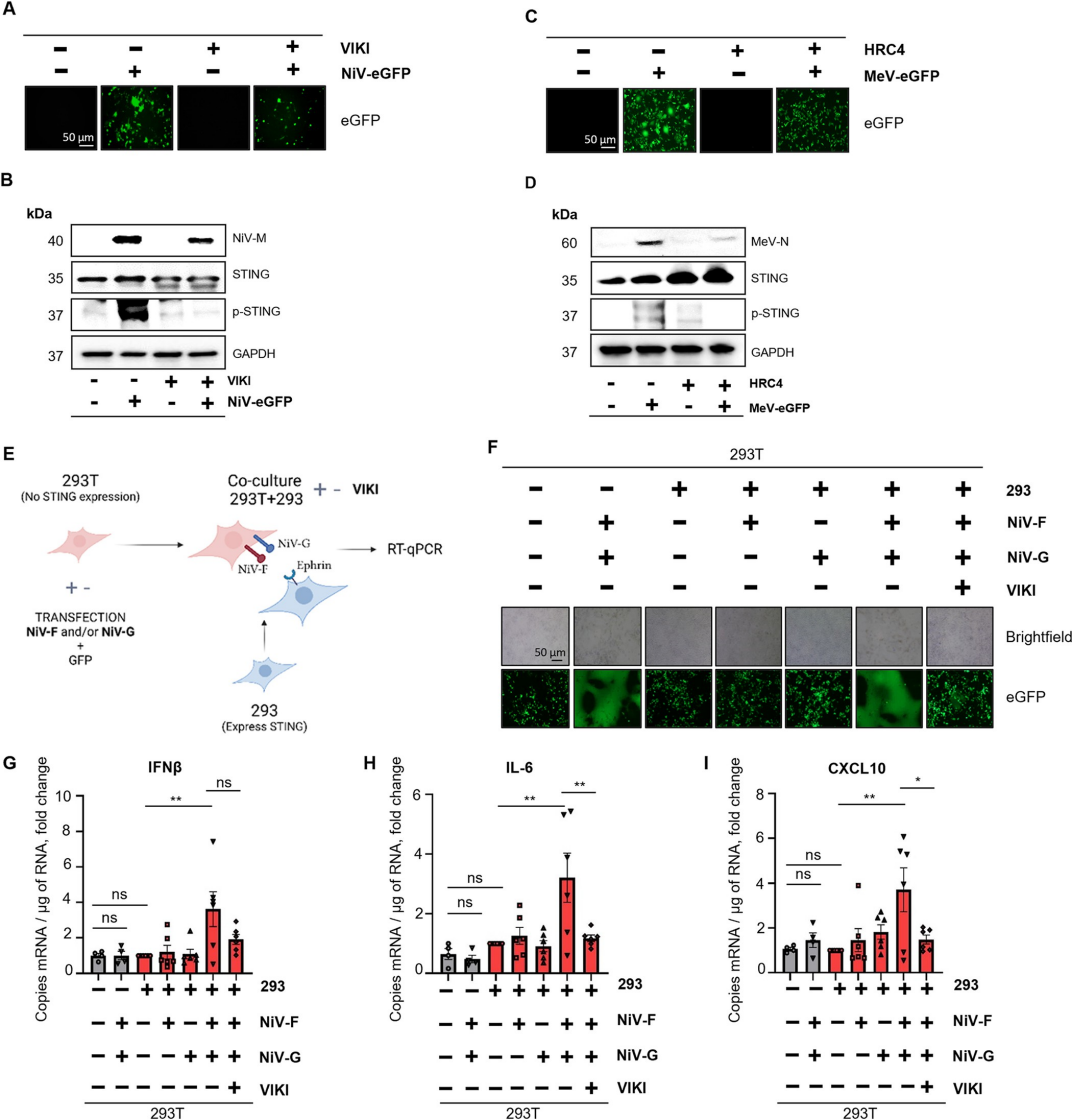
Viral-induced syncytia lead to STING activation and expression of IFN-I and inflammatory cytokines. (A-F) HPMEC cells were infected with NiV-eGFP at a MOI of 3 (A-B) or MeV-eGFP at a MOI of 1 (C-D) for 48h and treated or non-treated with VIKI or HRC4 fusion inhibitor peptides, respectively, at 2 μM 6h post infection. (A, C) eGFP expression was evaluated by fluorescence microscopy on NiV- (A) or MeV- (C) infected cells. (B, D) Cells were analyzed for NiV-M or MeV-N, STING, p-STING and GAPDH expression by western blot analysis (B, D). (E-I) 293T cells were treated or non-treated with VIKI at 1 μM, transfected with NiV-F and/or NiV-G and eGFP in presence or absence of empty vector and incubated overnight. The scheme was created with Biorender (agreement number: UA26WU6I2T) (E). 293T cells were then washed with PBS and co-cultured with 293 cells for 24h before undergoing RT-qPCR analysis. (F) eGFP expression and syncytia formation were evaluated by fluorescence microscopy. (G-I) Cells were harvested and analyzed by RT-qPCR for IFNβ, IL-6 and CXCL10 expression. Data from 293T (n = 4) and co-culture of 293T and 293 (n = 6) are represented as mean ± SEM and expressed as fold change compared to co-culture + empty vector condition. All samples were analyzed using one-way analysis of variance, followed by Tukey’s multiple comparison test, *p<0.05; **p<0.01; ***p<0.001; ****p<0.0001.

Since STING activation occurs during *Paramyxovirus* infections in the absence of a viral DNA agonist, we hypothesized that virus-induced perturbations of cellular homeostasis could represent a danger signal triggering STING axis through endogenous DNA, as observed in numerous other RNA virus and bacterial infections [35,45–52]. To identify a potential mechanism responsible for this phenomenon, we investigated whether syncytia formation could be associated with STING activation. To address its role, we infected HPMEC cells with NiV-eGFP (Fig 5A and 5B) or MeV-eGFP (Fig 5C and 5D) and cells were mock-treated or treated with virus-specific fusion inhibitory peptides so called VIKI-PEG4-Chol (VIKI), derived from Human Para-influenza virus 3, and HRC4, derived from MeV, respectively, 6 h post infection, thus allowing cell infection but preventing virus-induced cell-to-cell fusion [53–55] (Fig 5A and 5C). p-STING was strongly increased in infected mock-treated cells as observed by western blot analyses, while STING phosphorylation was reduced in infected cells treated with fusion inhibitory peptides (Figs 5B, 5D, S2A and S2B), suggesting that syncytia formation could be responsible for STING activation during NiV and MeV infection.

To analyze whether the sole viral syncytia formation is sufficient for an induction of STING response, in the absence of other virus-induced stress factors, we transfected 293T cells, that do not express STING or IFI16 and display weak cGAS levels, with plasmids coding for NiV-F and/or G envelope glycoproteins (S2C and S2D Fig) and eGFP. Then, 293T were self-cultured or co-cultured with 293 cells expressing all proteins involved in STING pathway (Figs 5E and S2D). First, we observed that despite syncytia formation in 293T cultures, no cytokine induction was observed (Fig 5F–5I). Then, we demonstrated that despite co-culture between 293T and 293 cells, no increase of IFNβ, IL-6 and CXCL-10 mRNA expression occurred in any condition that did not present syncytia, including mock-, single NiV F- or single NiV-G-transfected 293T cells (Fig 5F–5I). Finally, our results highlighted that syncytia formation detected following coculture of double NiV F/NiV G-transfected 293T with 293 cells triggered the expression of IFNβ, IL-6 and CXCL-10 cytokines that were all inhibited in the presence of VIKI fusion inhibitory peptides (Fig 5F–5I). Overall, our data proved that cell-cell fusion perpetrated by NiV envelope glycoproteins is responsible for the STING axis-dependent induction of cytokine expression.

### STING axis pathway activation is associated with mitochondrial stress and DNA damage following paramyxovirus-induced syncytia formation

**Fig 6:**
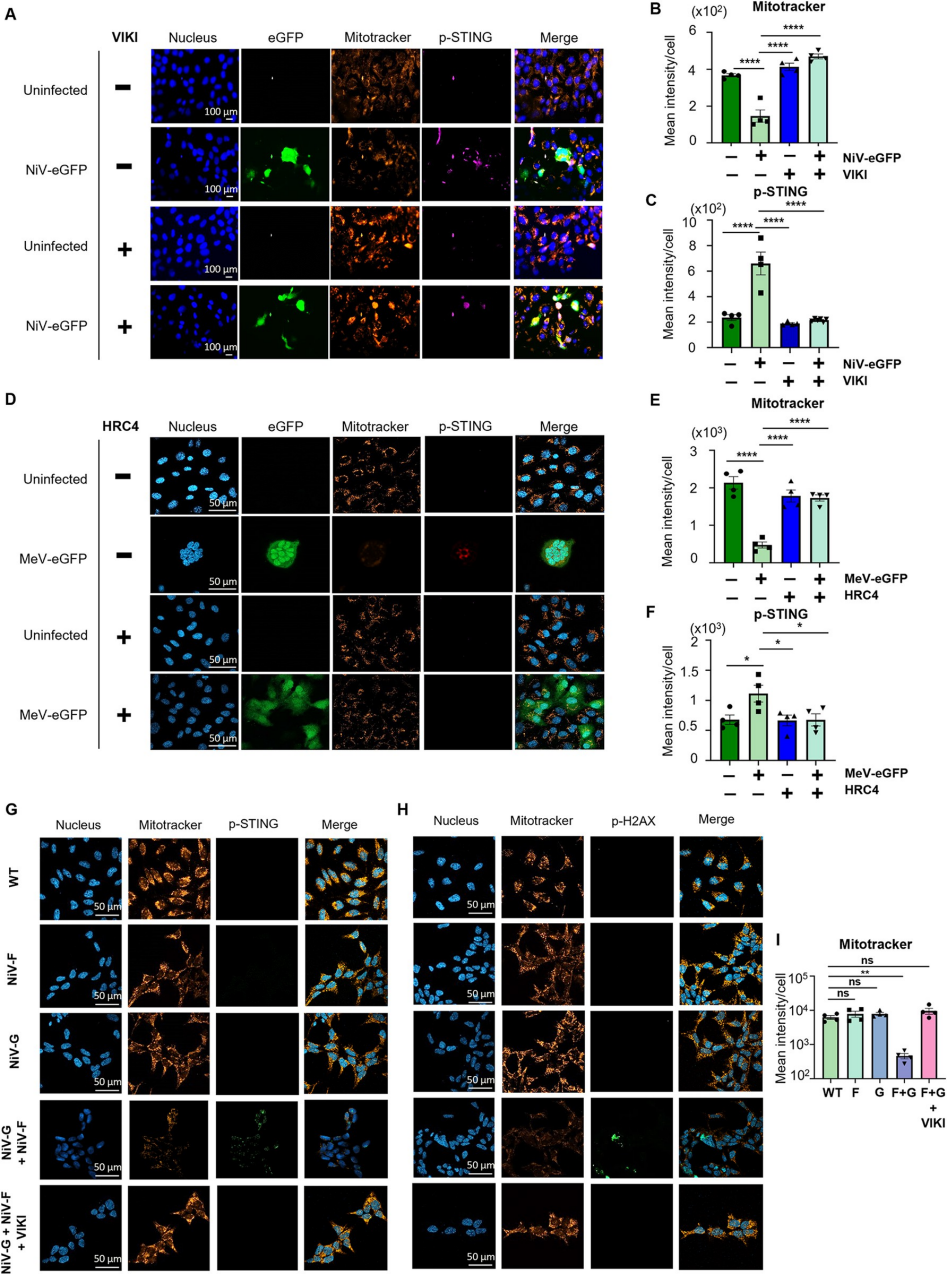
STING axis activation is associated with mitochondrial stress and DNA damage following paramyxovirus-induced syncytia formation. (A-F) HPMEC cells were infected with NiV-eGFP at a MOI of 3 (A-C) or MeV-eGFP at a MOI of 1 (D-F) for 48h and treated or non-treated with VIKI or HRC4 fusion inhibitor peptides, respectively, at 1 μM (n = 4). (A, D) NiV- (A) or MeV- (D) infected cells were stained with Mitotracker Orange 100 nM, fixed, stained with anti-p-STING antibody and analyzed by fluorescence microscopy. (B, C, E, F) The mean fluorescence intensity per cell of Mitotracker (B, E) or p-STING (C, F) staining was calculated using QuPath-0.4.3 on at least 50 events per condition from each of 4 independent replicates. For infected mock condition, only cells inside syncytia were analyzed. Data are represented as mean ± SEM. All samples were analyzed using one-way analysis of variance, followed by Dunnett’s multiple comparison test, *p<0.05; ****p<0.0001 compared to NiV- or MeV-infected WT condition. (G-I) HeLa cells WT or stably expressing NiV-F or NiV-G were cultivated individually or co-cultured, treated or non-treated with VIKI fusion inhibitor peptide at 1 μM and incubated for 48h (n = 3). Cells were stained with Mitotracker Orange at 100 nM, fixed, stained with anti-p-STING (G) or anti-phosphorylated histone 2AX (p-H2AX) (H) antibodies and analyzed by confocal microscopy. The mean fluorescence intensity per cell of Mitotracker staining (I) was calculated using QuPath-0.4.3 on at least 30 events per condition from each of 4 independent replicates. Data are represented as mean ± SEM. All samples were analyzed using one-way analysis of variance, followed by Dunnett’s multiple comparison test, *p<0.05; ****p<0.0001 compared to NiV- or MeV-infected WT condition.

We next aimed at identifying a potential intracellular perturbation responsible for STING pathway activation following NiV and MeV infection. As it was recently published that mitochondrial stress occurs during MeV infection leading to leakage of mitochondrial DNA (mtDNA) in the cytoplasm and further sensing by cGAS [35], we investigated whether mitochondrial dysfunction and STING activation could be associated to syncytia formation. HPMEC cells infected with NiV-eGFP (Fig 6A–6C) or MeV-eGFP (Fig 6D–6F) and treated or not with VIKI or HRC4 fusion inhibitory peptides, respectively, were stained with an anti-p-STING antibody and Mitotracker Orange CM-H2TMRos dye. Mitotracker is a mitochondrion-selective fixable live dye that penetrates in actively respiring cells, where it is oxidized and sequestered in mitochondria [56]. Infected cells displayed a significant decrease in Mitotracker intensity (Fig 6B and 6E), thus indicating a reduction in mitochondrial membrane potential that can be associated with mitochondrial dysfunction. In parallel, p-STING was detected in perinuclear areas in both NiV- and MeV-infected cells (Fig 6C and 6F). In contrast, Mitotracker and p-STING intensity in infected cells treated with fusion inhibitory peptides were equivalent to the non-infected condition (Fig 6B, 6C, 6E and 6F). These results show that both mitochondrial stress and STING activation occur in *Paramyxovirus*-induced syncytia.

To confirm that virus-induced cell-cell fusion is responsible for these observed effects, we cultured HeLa cells WT or stably expressing NiV-F or NiV-G envelope glycoproteins either alone or in co-culture in the presence or absence of VIKI fusion inhibitory peptides (Figs 6G–6I and S3). The correct expression of NiV-F and G was verified by flow cytometry (S3B Fig). Our results showed that Mitotracker intensity levels were reduced and p-STING levels were increased in the co-culture of HeLa-NiV-F and HeLa-NiV-G compared to WT HeLa and self-cultured HeLa-NiV-F or HeLa-NiV-G (Fig 6G and 6I). Moreover, these effects were reverted by the presence of fusion inhibitory peptides, thus confirming the involvement of paramyxovirus envelope glycoproteins (Fig 6G and 6I). Additionally, we observed in the co-culture an increased staining of the phosphorylated histone 2AX (p-H2AX), a marker of double strand breaks (DSB) indicating DNA damage (Fig 6H). Moreover, relocalization of both nuclear and cytoplasmic cGAS to perinuclear areas was observed (S3C and S3D Fig). In parallel, translocation of IFI16 from nucleus to cytoplasm was observed in some infected cells, even though the major IFI16 pool remained mostly nuclear (S3E and S3F Fig). All observed effects were prevented in fusion peptide-treated conditions (Figs 6G, 6H and S3C–S3F). Overall, these results demonstrate that viral-induced syncytia trigger mitochondrial stress, associated with DNA damage and the presence of both cGAS and IFI16 perinuclear location as confirmed by the analysis of fluorescence spectra (S3D and S3F Fig).

### Mitochondrial DNA is responsible for STING activation through its sensing by both cGAS and IFI16 during NiV and MeV infection

**Fig 7:**
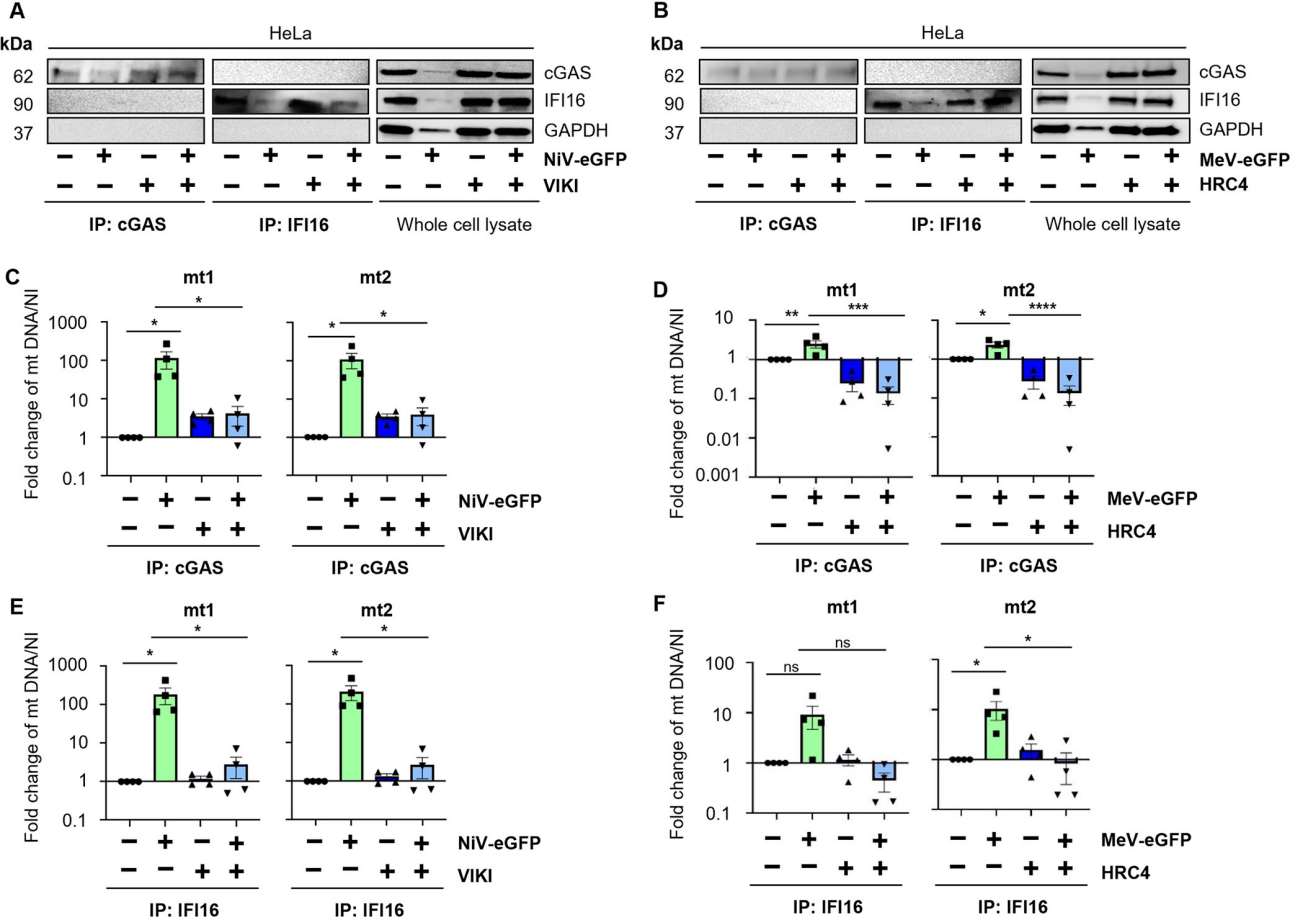
Mitochondrial DNA is responsible for STING activation through its sensing by both cGAS and IFI16 during NiV and MeV infection. HeLa cells were infected with NiV-eGFP (A-C) or MeV-eGFP (D-F) at a MOI of 0.3 and treated or not with VIKI or HRC4 fusion inhibitor peptides, respectively, at 1 μM 6h post infection (n = 4). 48h post infection, cytoplasm was extracted and cGAS and IFI16 proteins were immunoprecipitated from cytoplasmic extract. DNA was purified from the immunoprecipitated products and analyzed by qPCR using primers specific for mtDNA. (A,B) Immunoprecipitated cGAS and IFI16 and whole cell lysates from NiV- (A) and MeV- (B) infected cells were analyzed for cGAS, IFI16 and GAPDH expression by western blot. (C-F) Purified DNA from immunoprecipitated cGAS (C, D) or IFI16 (E, F) was analyzed by qPCR for two mitochondrial (mt1 and mt2) DNA regions. Data are represented as mean ± SEM. All samples were analyzed using one-way analysis of variance, followed by Dunnett’s multiple comparison test, *p<0.05; ***p<0.001; ***p<0.0001 compared to NiV-infected WT condition.

**Fig 8:**
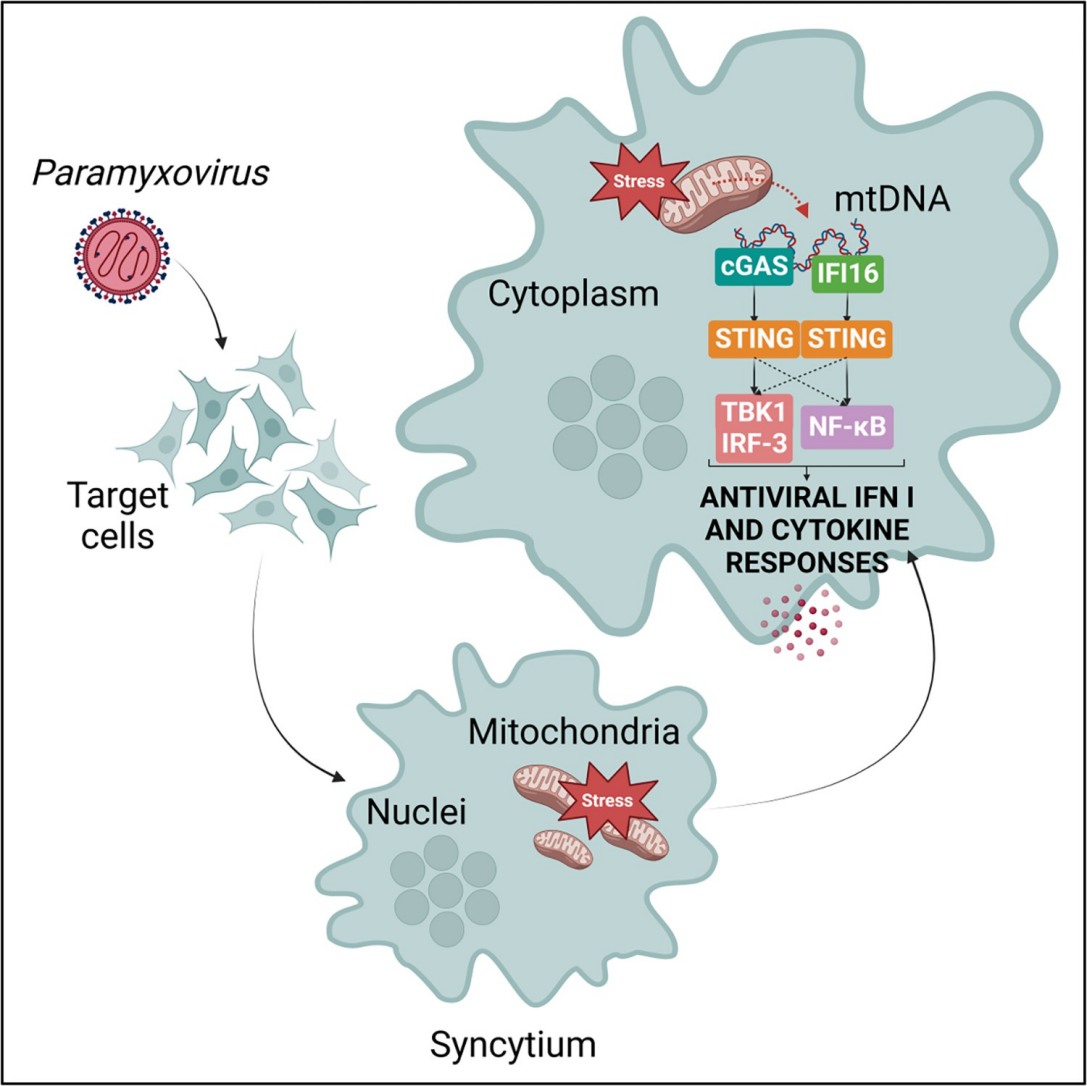
Schematic model representing the mechanisms responsible for STING activation following Nipah and Measles virus infection. *Paramyxovirus* infection provokes the structural rearrangement of target cells into multi-nucleated giant cells (syncytia), leading to mitochondrial stress induction. As a consequence of mitochondrial stress, mitochondrial DNA (mtDNA) is released in cytoplasm and detected by both cGAS and IFI16 intracellular DNA sensors. While cGAS preferentially activates STING/TBK1/IRF-3 axis leading to IFN-I response induction, IFI16 primarily activates STING/NF-κB and inflammatory cytokines response, which may altogether play a role in the antiviral protection. The scheme was created with Biorender (agreement number: HC26WU6FDX).

We finally investigated intracellular agonists that could be involved in the activation of STING axis. As previously shown, cGAS detects cytosolic mtDNA during MeV infection [35], thus we extended our analysis to NiV and considered the potential role of IFI16. To study whether IFI16 contributes to the detection of cytoplasmic endogenous DNA in parallel to cGAS following syncytia formation, we performed a co-immunoprecipitation experiment on WT HeLa cells infected with NiV-eGFP (Figs 7A, 7C, 7E and S4A) or MeV-eGFP (Figs 7B, 7D, 7F, S4A and S4B) in the presence or absence of VIKI or HRC4 fusion inhibitory peptides, respectively. Both cGAS and IFI16 proteins were immunoprecipitated from cytoplasm extracts (Fig 7A and 7B) and potential DNA bound to cGAS and IFI16 was analyzed by qPCR with mtDNA- or nuclear DNA-specific primers (Figs 7C–7F, S4C–S4H and S5). First, we analyzed cytoplasmic and nuclear protein expression profiles by western blot (S4B Fig) and verified that our selected target genes were amplified by our couples of primers in HeLa cells by qPCR (S4C Fig). After IP analysis, a significant increase in mtDNA co-immunoprecipitated with cGAS was measured following NiV and MeV infection (Fig 7C and 7D), confirming previous observations [35]. In addition, immunoprecipitated IFI16 as well resulted to be enriched in mtDNA following infection (Fig 7E and 7F). Moreover and concomitant to previous data, no nuclear DNA was detected neither in cGAS nor in IFI16 immunoprecipitation (S4E–S4H Fig) [35]. To exclude that the lack of nuclear DNA detection was due to technical issues, we immunoprecipitated tri-Methyl-Histone H3, a well-known nuclear DNA-binding protein, and we detected co-immunoprecipitated nuclear DNA and not mitochondrial DNA (S4D Fig). Furthermore, the levels of mtDNA co-immunoprecipitated with cGAS and IFI16 were significantly reduced in infected cells treated with fusion inhibitor peptides compared to non-treated infected condition (Fig 7C–7F). Altogether, these results strongly suggest that, following *Paramyxovirus* infection and the subsequent syncytia formation, mtDNA is released in cytoplasm and represents the main cellular agonist detected by both cGAS and IFI16 and responsible for the activation of STING axis (Fig 8).

## Discussion

Our previous work highlighted that DNA-sensing STING pathway is involved in the response against NiV and MeV infection [15,16]. However, the precise role of STING and the cellular mechanisms responsible for its activation during *Paramyxovirus* infection remained obscure and needed further investigations.

To clarify the role of STING *in vivo*, we infected WT, IFNAR KO and STING KO mice with NiV and monitored animal survival, viral replication and cytokine expression for 28 days. We observed that the absence of STING alone did not impair animal survival, as in STING KO mice TLR and RLR signaling pathways which are primarily responsible for viral RNA detection remained fully expressed. However, STING deletion resulted in a higher viral load and lower CXCL10 expression in KO animals compared to WT mice. Furthermore, a compromised STING response could have an impact on humoral adaptive immunity, as previously described [57,58], as suggested by the fact that STING KO mice displayed lower neutralizing antibodies titers compared to WT mice. As a consequence, it would be interesting to test whether a treatment with specific STING agonists, alone or as vaccine adjuvant, could contribute to boost both innate and adaptive immune responses against NiV infection [59].

While we previously focused on the activation of IFN-I production, we later observed that both IFN-I and NF-κB responses are induced in a STING-dependent manner after NiV infection. Interestingly, cGAS appeared to be more important in the activation of IFN-I rather than NF-κB, leading us to hypothesize the presence of an alternative DNA sensor mainly involved in the trigger of STING-dependent NF-κB signalling in parallel to cGAS. As IFI16 was demonstrated to initiate non-canonical STING activation mainly through ubiquitination followed by subsequent NF-κB induction, in opposition to the canonical cGAS/cGAMP/STING/IRF3 pathway, we tested the implication of IFI16 in the immune response to *Paramyxovirus* infection [40]. We observed that IFI16 is involved in the control of viral replication following both NiV and MeV infection and has a major effect on NF-κB p65 phosphorylation and IL-6 expression downstream STING. Thus, these results confirm that both cGAS and IFI16 contribute to trigger a STING-dependent response following *Paramyxovirus* infections, as previously observed, and further studies could be useful to dissect the impact of each sensor on post-translational modifications associated to STING and their effects on the downstream transcriptional response. Indeed, it remains obscure whether these two axes are independent or if there is competition, synergy or crosstalk between them, as contrasting results have been published, with striking differences according to the activating stimuli and cellular models [40,60–65]. For example, it has been shown that IFI16 can contribute to IFN-I induction through the activation of STING and TBK1 to enhance cGAMP-dependent activation of STING as described in THP-1 cells following HSV-1 and HIV infection, however it is unknown whether the same phenomenon is induced by NiV and MeV [63]. Understanding the interaction among the two pathways would be of great interest, as they could play an important role in antiviral immune regulation.

To decipher viral-dependent cellular mechanisms associated to STING activation we addressed the involvement of syncytia, the most important cytopathic effect perpetrated by both NiV and MeV, as the formation of multi-nucleated giant cells had been previously associated to the non-canonical activation of STING [49,50]. We observed that virus-induced cell-cell fusion triggers the phosphorylation of STING *in vitro*, supporting the previous finding that MeV-induced fusion amplifies IFN-I response [66]. However, we cannot exclude that additional virus-induced danger signals could contribute to STING activation, with possible differences according to viral strains and/or target cell types. Moreover, it was previously described that MeV infection induces mitochondrial stress through the downregulation of mitochondrial biogenesis and that V protein of MeV can damage the mitochondrial network by interacting with delta-aminolevulinate synthase 1 (ALAS1) [35,67]. We confirmed in this work that a reduction in mitochondrial membrane potential occurs following both NiV and MeV infection and we extended our demonstration that it is triggered through a fusion-dependent process. Nonetheless, the mechanism linking syncytia formation to mitochondrial stress remains unknown and further investigations are required. Moreover, we showed here that STING phosphorylation is triggered in THP-1 monocytic cell line despite minimal syncytia formation. This could be due to the co-existence of multiple mechanisms of STING activation with possible variations according to different cell types.

Finally, we observed that, following NiV and MeV infection, mtDNA is released in the cytoplasm through a syncytia-dependent mechanism and sensed by both cGAS and IFI16, confirming that STING is both canonically and non-canonically activated by endogenous DNA during *Paramyxovirus* infection. Despite the fact that nuclear DNA was not detected along mtDNA released in the cytoplasm, we cannot exclude that genomic DNA might participate in STING activation through uncharacterized mechanisms. Further analyses on physiological cellular targets of NiV and MeV infection or *in vivo* would corroborate these observations and clarify the role of nuclear DNA. Moreover, while NiV infection induced a 100-fold increase of mtDNA co-immunoprecipitated with cytoplasmic cGAS and IFI16 compared to non-infected cells, only a less-than 10-fold mtDNA enrichment was observed following MeV infection, which could lead to a different potency of STING activation and possible consequences on inflammatory cytokine and IFN-I production. This could be due to the fact that viral loads in non-treated NiV-infected samples were higher compared to treated infected samples, while these differences were not observed in MeV-infected cells (S4A Fig), underlying a different speed in viral replication *in vitro* between these two paramyxoviruses.

It is interesting to note that NiV and MeV induce the activation of the STING axis in human cells despite the fact that this signaling pathway is detrimental for viral growth. Moreover, differently from what was observed for other RNA viral families, such as *Flaviviridae* and *Coronaviridae* [13], no viral protein of NiV has been shown to antagonize the STING axis yet. This could be a consequence of the fact that humans represent an accidental host for NiV, whose natural reservoir is the fruit bat. Bats have a dampened STING response, which prevents a STING-dependent hyper-inflammation that would derive from the high amount of cytosolic DNA generated through metabolic stress during flight activity [68]. This could contribute to explain the ability of these mammals to host NiV, which is highly pathogenic for humans, without developing symptoms. Thus, it would be interesting to compare human and bat STING pathway responses following *Paramyxovirus* infection to clarify a possible link between STING over-activation and pathogenicity in humans.

It has been demonstrated that other RNA viruses, such as Dengue virus, trigger STING activation through mitochondrial damage induction [46], or through the generation of syncytia, as observed in SARS-CoV-2 infection [50], but no connection between syncytia formation and mitochondrial damage was established before our work. We suggest that it could be useful to test these mechanisms in the context of other RNA virus infections to understand whether this could represent a conserved pattern of STING activation.

In conclusion, we demonstrate that during NiV and MeV infection, the STING pathway is activated through the recognition of leaked mtDNA detected by both cGAS and IFI16 following the formation of syncytia, leading to the subsequent expression of both IFN-I and inflammatory cytokines. Its important role in the control of NiV and MeV infection *in vitro* and *in vivo* suggests the credible employment of STING-targeting molecules as promising therapeutic strategies to treat *Paramyxovirus* infections.

## Materials and methods

### Ethics statement

Animal experiments in the BSL-4 of Budapest were performed according to the guidelines of the European Communities Council Directive (86/609 EEC) and were approved by the Hungarian National Authority (Scientific Ethics Council for Animal Experiments, PE/EA/1456-7/2020).

Animals in transit at Plateau de Biologie experimentale de la souris (PBES) in Lyon were manipulated in accordance to good experimental practice and approved by the regional ethics committee CECCAPP (Comité d’Evaluation Commun au Centre Léon Bérard, à l’Animalerie de transit de l’ENS, au PBES et au laboratoire P4) and authorized by the French Ministry of Higher Education and Research (no. 00962.01).

### Mice

Wild type (WT) C57BL/6J mice (Charles River), B6.129S2-Ifnar1tm1(Neo)Agt (IFNAR-KO) [69] and B6(Cg)-Tmem173tm1.2Camb/J (Tmem173−/−, STING KO) [70] mice were bred under specific pathogen-free conditions in the central mouse facility of the Helmholtz Centre for Infection Research, Brunswick, and at TWINCORE, Centre for Experimental and Clinical Infection Research, Hanover, Germany. Mouse experimental work was carried out using 4-week-old to 6-week-old mice and sex balance between male and female was respected.

### Cell lines

Human monocytic THP-1 cell lines WT, cGAS KO, STING KO or IFI16 KO were obtained from Veit Hornung lab [71] and cultured in RPMI 1640 GlutaMAX supplemented with 10% heat-inactivated FBS, 1% HEPES and 1% penicillin-streptomycin mix. For infection, THP-1s were plated in 12-well plates at 5x10^5^ cells/well and cultured with rNiV-Malaysia-eGFP (NiV-M-eGFP) at a MOI of 0.3 or 1 or rMeV-EdmH-eGFP at a MOI of 0.1 or 1 for 48h.

Human pulmonary microvascular endothelial cells (HPMEC) [72] from a male donor were cultured in Endothelial Cell Growth Medium GM MV2 (Promocell) and SupplementalPack (5% FCS, 5 ng/ml recombinant hEGF, 10 ng/ml recombinant human bFGF, 20 ng/ml long R3 IGF, 0.5 ng/ml recombinant hVEGF, 1 μg/ml ascorbic acid), in flasks coated with 0.1% bovine gelatine (Sigma) in PBS. For infection, HPMECs were plated at 2.5x10^5^ cells/well in 12-well plates or at 5x10^4^ cells/well in 8-well Ibidi slides and cultured with NiV-M-eGFP at a MOI of 1 or 3 or MeV-EdmH-eGFP at a MOI of 1 PFUs/cell for 48 h.

293T cells (ATCC) were cultured in DMEM supplemented with 10% FBS, 1% HEPES and 1% penicillin-streptomycin. For transfection, 293T were plated in 24-well plates at 5x10^5^ cells/well. 16 h after transfection, 293T cells were co-cultured with 293 cells (ATCC) in 12-well plates for 24 h.

WT and phCMV-NiV-G or phCMV-NiV-F-transfected human adenocarcinoma epithelial cells (HeLa) were cultured in DMEM supplemented with 10% FBS, 1% HEPES and 1% penicillin-streptomycin. HeLa WT cells were plated in 6-well plates at 5x10^5^ cells/well and then transfected with 2 μg phCMV.NiV-G or phCMV.NiV-F generated as previously described [73]. Then cells were selected using G418 at 1 mg/ml before being cultured as described above. For microscope analysis, HeLa cells (ATCC) were plated in 8-well Ibidi slides at 4x10^5^ cells/well and cultured for 48h before fixation. For immunoprecipitation, WT HeLa cells were plated in 6-well plates at 2.5x10^5^ cells/well and cultured with NiV-M-eGFP or MeV-EdmH-eGFP at a MOI of 0.3 for 48h. All cell types were incubated at 37°C with 5% CO2 and were tested negative for Mycoplasma spp.

### Viruses

NiV-Mal (isolate UMMC1 GenBank- AY029767), recombinant NiV (rNiv)–enhanced green fluorescent protein (eGFP) [74] and recombinant MeV IC323 vaccine strain, expressing Edmonston H and eGFP [75], kindly provided by Dr Y. Yanagi (Kyushu University, Japan) and were prepared by infecting Vero-E6 cells (ATCC), in the INSERM Jean Mérieux biosafety level 4 (BSL-4) and BSL-2 laboratories at CIRI in Lyon, France respectively.

### Intraperitoneal infection of mice

All work with live Nipah Virus Malaysian Strain (NiV-Mal) was performed under BSL-4 conditions at the National Biosafety Laboratory at the National Center for Public Health and Pharmacy in Budapest, Hungary. All mice were inoculated intraperitoneally with 10^6^ TCID50 NIV-Mal under inhalational anesthesia with isoflurane (isoflurane: ISOFLUTEK 1000 mg/g, Laboratorios Karizoo S.A.; anesthesia station: MiniHUB-V3, TEM SEGA, France).

### Sample collection and preparation

Euthanasia with CO2 was conducted under inhalational anesthesia with isoflurane as well, followed by blood collection via cardiac puncture, and autopsy. During autopsy collection of brain, lung and spleen tissue were performed for further nucleic acid isolation and immunohistochemistry. Organs were divided for molecular testing and were collected and freezed immediately on -80oC without any medium until further preparation. Parts of organs for immunohistochemistry were collected into fixative 10 V/V% formaldehyde-PBS solution. Then, 25–30 mg of the thawed organs were taken into MagNA Lyser Green Beads tubes (Roche Diagnostics GmbH, Mannheim, Germany) containing 600 μL of 1:101 mixture of β-mercaptoethanol and Buffer RLT (QIAGEN GmbH, Hilden, Germany). After homogenisation using MagNA Lyser instrument (Roche Diagnostics GmbH), samples were centrifuged for 5 minutes at 12000 rpm. Supernatants were transferred into new tubes and after 10 minutes of incubation at room-temperature an equal volume of 70 V/V% ethanol was added into each tube.

### Seroneutralization

Seroneutralization assay was performed on a selected serum sample set as previously described62. Sera were diluted in 7-point serial twofold dilution in triplicate in serum-free Dulbecco’s Modified Eagle’s Medium (DMEM, VWR) in sterile 96-well microtiter plates. Positive and negative control sera were also applied (with and without serum for negative control). An equal volume of 60±20 TCID50 NiV-Mal was added into each well and incubated for 1 hour on 37oC. After this step samples were transferred onto monolayer Vero E6 cells (approximately 1.8E+05 cells/mL) maintained in serum-free DMEM in 96-well cell culture plates (TPP, Switzerland). The neutralizing antibody titers of each sample were determined by the lack of cytopathic effect (CPE) after a 5 days incubation in a 37oC CO2 incubator. For triplicates the geometric mean of end dilutions was calculated and reported as a neutralizing antibody titer (Nab).

### Drugs

H-151, a specific inhibitor for STING (InvivoGen, Cat# inh-h151) and RU.521, a specific inhibitor for cGAS (InvivoGen, Cat# inh-ru521), were added 1 h before infection of HPMEC cells at 10 μM and 10 μg/ml, respectively, selected according to the previously published results [76,77]. Then, cells were infected with rNiV-eGFP and incubated for 48 h at 37°C with 5% CO2.

### Fusion inhibitory peptides

Unconjugated MeV HRC peptide was purchased from Shanghai Ruifu Chemical Co., Ltd. Bis-maleimide cholesterol was custom made by Charnwood Molecular, Ltd. HRC4 was conjugated with cholesterol and purified as previously described [55,78].

VIKI-PEG4-Chol fusion inhibitor peptide was produced by standard Fmoc-solid phase methods and cholesterol was attached to peptides as previously described 49. Polyethylene glycol (PEG) was obtained from Quanta BioDesign (Plain City, OH).

Virus-specific fusion inhibitory peptides were diluted in culture medium and added 6h post infection or in correspondence to transfection at 2 μM or 1 μM.

### RNA extraction and RT-qPCR

For mouse samples, total nucleic acid extraction was performed using QIAsymphony SP instrument with QIAsymphony DSP Virus/Pathogen Mini Kit and Complex200_OBL_V4_DSP protocol (QIAGEN GmbH, Hilden, Germany).

For in vitro experimentations, cells were collected at indicated time points and RNA extracted using appropriate NucleoSpin RNA Kits according to the manufacturer’s instructions.

Equal amounts of extracted RNA (100 ng) were reverse transcribed using the iScript Select cDNA Synthesis Kit and amplified by real-time PCR using Platinum SYBR Green qPCR SuperMix-UDG on a StepOnePlus Real-Time PCR System. Pfaffl Model [79] and Bustin MIQE checklist [80] were used for all validations and calculations. To validate the efficacy of our primers, a PCR on a positive control (cDNA from corresponding stimulated cells) has been performed before dosing several dilutions of our PCR products using the Denovix DS-11-FX spectrophotometer to evaluate the quantity of DNA in each dilution. Then, the PCR products were 10-fold serially diluted with a range from 10–1 to 10–12 to validate efficacy, specificity and sensitivity of each couple of primers used for qPCR. Moreover, the exact number of copies in each qPCR reaction was obtained by calculating the “N0 Samples = N0 Standard * Efficacy-ΔCt” before being converted using molecular weight of each targeted gene and Avogadro number. Results obtained were converted to “copies/μg of RNA” for cell lysates. Finally, normalization was performed by dividing obtained numbers of RNA copies of the target genes with the deviation of the glyceraldehyde 3-phosphate dehydrogenase (GAPDH) used as house-keeping gene. Specific sets of primers were designed and validated for the detection of human hGAPDH, hIFNβ, hCXCL-10 and hIL-6, murine mGAPDH, mCXCL-10 and mIFNβ and viral NiV-N and MeV-N.

### Immunohistochemistry

Brain, lung and spleen from mice were embedded in paraffin wax and sectioned at 7 μm. Slides were deparaffinated and rehydrated in three Xylene baths for 5 min each, followed by two 100% alcohol baths for 5 min, and then succeeded with multiple baths using decreasing level of alcohol for 3 min each. After deparaffination, slides were put in a sodium citrate solution in a boiling water bath for 20 min for heat-induced epitope retrieval and washed 3 times in PBS for 3 min afterwards. Activity of endogenous peroxydase was blocked using a H2O2 0.3% solution. Blocking of non-specific epitopes is done using PBS-2.5% decomplemented Normal Horse Serum + 0.15% Triton X-100 for 30 min. Then, primary rabbit anti-NiV N antibody was used at 1/10000 dilution and incubated overnight at 4°C in the blocking buffer. For secondary antibody and revealing steps, ImmPress system (anti-rabbit ig/peroxydase) was used. Counterstaining was performed using Harris solution and photographs were taken with a microscope Zeiss Axiovert 100M.

### Flow cytometry

THP-1 cells were seeded in 12-well plates at 2.5×105 cells/well before being infected with NiV-eGFP and MeV-eGFP at a MOI of 0.3 or 0.1, respectively, and cultured for 48 h at 37°C with 5% CO2. Then, cells were washed with PBS 1X and fixed with methanol-free paraformaldehyde (PFA) 4% 15 minutes at 4°C. Afterwards, cells were reconstituted in PBS 1X and evaluated for eGFP expression using a Gallios flow cytometer in the BSL-4 or a 4L Fortessa flow cytometer. 293T transfected with or without NiV-F or NiV-G plated in 24-well plates and 293 cells prior to co-culture were detached by resuspension and transferred in polystyrene tubes. HeLa-NiV-F and HeLa-NiV-G cells were washed with PBS, detached with TrypLE 5–10 minutes at 37°C and transferred to polystyrene tubes after inactivating TrypLE with culture medium. 293T, 293 and HeLa were then washed 4 minutes at 1500 rpm with PBS 1X and incubated with primary anti-NiV-F or anti-NiV-G antibodies in PBS-1% fetal bovine serum (FBS) 30 minutes at 4°C. Cells were washed again before being incubated with II anti-mouse AF488-conjugated antibody in PBS-1% FBS 20 minutes at 4°C. Following two washes in PBS 1X, AF488 expression was measured using a 4L Fortessa flow cytometer.

### Fluorescence microscopy

Following infections of THP-1 and HPMEC cells with NiV-eGFP and MeV-eGFP or transfection of 293T cells with eGFP, GFP fluorescence was visualized at Leica DMIRB microscope in BSL-4 or Eclipse Ts2R NIKON microscope in BSL-2.

### Immunoblot analysis

Heated protein lysates were separated by SDS-PAGE in Any kD Mini-PROTEAN TGX Precast Protein Gels (Bio-Rad) and electro transferred for 7 minutes onto polyvinylidene difluoride (PVDF) membranes using Trans-Blot Turbo Transfer System and Trans-Blot Turbo Midi 0.2 μm PVDF Transfer Packs (Bio-Rad). PVDF membranes were blocked in Tris-buffered saline containing 10% milk for 1 h at room temperature and then incubated overnight at 4°C with the following primary antibodies diluted 1:1000 in TBS-0.1% Tween-0.5% milk: mouse anti-GAPDH (Millipore), rabbit anti-STING, rabbit anti-S366 p-STING, rabbit anti-p65, rabbit anti-phospho-p65, rabbit anti-cGAS, rabbit anti-tri-methyl-histone H3 (all Cell Signaling) and mouse anti-IFI16 (SCBT). Membranes were then washed 3 times using TBS-0.1% Tween and incubated 1 h at room temperature with horseradish peroxydase conjugated anti-mouse or anti-rabbit IgG antibodies (1:5000) or Trueblot antibodies (1:1000) diluted in TBS-0.1% Tween-0.5% milk. Membranes were then washed 3 times in TBS-0.1% Tween, once in TBS and incubated in Super Signal West Dura or in Super Signal West Femto reagent (Thermoscientific) for 1–3 minutes. Chemiluminescent signals were measured with the ImageQuant LAS 4000 Imaging System.

### Transfection and co-culture

293T cells were plated in 24-well plates at 5x105 cells/well and transfected with a mix of NiV-Mal-F and/or NiV-Mal-G, pCAGGS empty vector and eGFP plasmids using TransIT-LT1 kit according to the manufacturer’s instructions. Right after transfection, cells were treated or not with VIKI fusion inhibitor peptide at 1 μM and incubated overnight at 37°C and 5% CO2. 293T were then washed twice with PBS 1X to eliminate residual plasmids in the supernatant and co-cultured with 293 cells (5X105 cells/well) in 12-well plates for 24h prior to RNA extraction and qPCR.

### Immunofluorescence

For HPMEC infections, 5x104 cells were seeded in 8-well Ibidi slides before being infected with NiV-eGFP or MeV-eGFP at a MOI of 2 or 1, respectively, and cultured for 48 h at 37°C with 5% CO2. For HeLa WT or HeLa-NiV-F and HeLa-NiV-G coculture, cells were plated at 4x104 cells/well in 8-well Ibidi slides and incubated 48 h at 37°C with 5% CO2. Cells were washed with PBS and stained with Mitotracker Orange 100 nM according to the manufacturer’s instructions before undergoing fixation with PFA 4% 20 minutes at 4°C. Slides were permeabilized and blocked with a PermBlock solution (3% bovine serum albumin (BSA)-0.3% Triton X-100 in PBS 1X) for 30 minutes and incubated overnight at 4°C with the following primary antibodies: rabbit anti-phospho-STING (Cell Signaling), rabbit anti-phospho-histone H2A.X (Cell Signaling), mouse anti-cGAS (SCBT) or mouse anti-IFI16 (SCBT) diluted 1:100 in PermBlock solution. Slides were then washed 3 times with PBS 1X, incubated 1 h at room temperature with a mix of fluorophore-conjugated secondary antibody (anti-rabbit AF647, anti-rabbit AF488 or anti-mouse AF488 all from Invitrogen) 1:750 and DAPI (Merck) 1:1000 in PermBlock. Finally, slides were washed 5 times with PBS 1X, covered with Fluoromount-G mounting medium (Invitrogen) and stored at 4°C. Fluorophore expression was evaluated using a Zeiss Axio OBSERVER Z.1 microscope in the BSL-4 or a Confocal ZEISS LSM800 microscope, and photographs were treated using ImageJ software version Java 1.8.0_112.

### Cytoplasm extraction and immunoprecipitation

HeLa cells were plated in 6-well plates at 2.5x105 cells/well and infected with NiV-eGFP or MeV-eGFP at a MOI of 0.3. 6 hours post infection, cells were treated or not with VIKI or HRC4 fusion inhibitor peptides, respectively and incubated 48 h at 37°C with 5% CO2. Cells were washed with PBS 1X and detached with trypsin prior to cytosol extraction, which was performed using a CE buffer (HEPES 10 mM, KCl 60 mM, EDTA 1 mM, NP-40 0.075% V/V, DTT 1 mM and protease-phosphatase inhibitor 1 mM in PBS) adjusted to pH 7.6 according to Rockland’s cytoplasm and nucleus extraction protocol (https://www.rockland.com/resources/nuclear-and-cytoplasmatic-extract-protocol/). 100 μl of samples were kept to perform western blot analysis. 500 μl of RIPA and protease-phosphatase inhibitor solution were added to whole cell, cytoplasmic or nuclear fractions, which were lysed in rotation 30 minutes at 4°C and centrifuged 10 minutes at 10000 rpm at 4°C. The supernatants were incubated with rabbit anti-cGAS antibody (Cell Signaling) 1:100, mouse anti-IFI16 antibody (SCBT) 1:100, rabbit anti-tri-methyl-histone H3 antibody (Cell Signaling) 1:50 or normal rabbit IgG (Cell Signaling) 1:50 2 h in rotation at 4°C. EZview Red agarose beads were added to the lysates and samples were incubated overnight at 4°C in rotation. Beads were pulled down by centrifugation 1 minute at 10000 rpm at 4°C and washed 3 times with RIPA buffer on ice. From each tube, 125 μl of immunoprecipitated product were mixed with LDS and reducing agent for western blot analysis and 375 μl underwent DNA extraction.

### DNA extraction and qPCR

Immunoprecipitated agarose beads were washed with PBS 1X. 500 μl of NTI buffer from NucleoSpin Gel and PCR Clean-up kit (Macherey-Nagel) were added before heating samples 1 h at 60°C and DNA extraction was then performed according to manufacturer’s instructions. DNA was then analyzed by qPCR using couples of primers specific for mitochondrial (mt) or nuclear (Nuc) DNA as described by Sato et al. [35]. The quantity of DNA was normalized on protein bands measured by densitometry, calculated as quantity of immunoprecipitated target over whole cell target over whole cell GAPDH. All values were expressed as fold change compared to the non-infected condition.

### PCR and sequencing

To verify the correct amplification of target mitochondrial and nuclear genes, the mitochondrial (mt 1 and mt 2) and nuclear (Nuc DNA 1 and Nuc DNA 2) were amplified from HeLa cells using Platinum II Hot-Start PCR Master Mix (2X) kit (Thermofisher) with the following PCR primers:

mt 1 F: CTATCACCCTATTAACCACTCA

mt 1 R: TTCGCCTGTAATATTGAACGTA

mt 2 F: CACCCAAGAACAGGGTTTGT

mt 2 R: TGGCCATGGGTATGTTGTTAA

Nuc DNA 1 (LAMA4) F: ATGGCTTTGAGCTCAGCCTG

Nuc DNA 1 (LAMA4) R: TGAGCTCCCTTCAATGTC

Nuc DNA 2 (JAK1) F: AGAGGACTGCAATGCCATGG

Nuc DNA 2 (JAK1) R: CCGACAGATAGAAGATCAC

PCR products were purified using NucleoSpin Gel and PCR Clean-up kit (Macherey Nagel) and analyzed by Sanger sequencing (Eurofins genomics, Köln) using forward and reverse primers separately. DNA sequence was retrieved from the chromatogram of one or both sequencing reactions using Ape [81]. Sanger sequences were manually aligned to the following reference sequences obtained from Genbank: Mt1 and Mt2: mitochondrion genome OR088596, Nuc1: laminin subunit alpha 4 NM_002290.5 and Nuc2: Janus kinase 1 NM_001321853.1.

### Quantification and statistical analysis

#### Flow cytometry analysis

The results are presented in the form of histograms representing the mean eGFP positive cells for each condition and error bars representing the standard error mean (SEM) for n = 6 experimental replicates. The different conditions were compared to the control (WT infected). Statistical significance was assessed by a one-way ANOVA, followed by a Tukey’s multiple comparisons test; *p < 0.05, **p < 0.01, ***p < 0.001 and ****p < 0.0001 (threshold of significance of 5%).

#### qPCR analysis

The results are presented in the form of histograms, representing the mean of copies of mRNA for a gene for each condition and error bars representing the standard error mean (SEM). Points represent the number of experimental replicates. Statistical significance was assessed by t-test or one-way ANOVA, followed by a Tukey’s multiple comparisons test; *p < 0.05, **p < 0.01, ***p < 0.001 and ****p < 0.0001 (threshold of significance of 5%).

#### Densitometry

Densitometric analyses of phospho-STING were performed using ImageJ 1.52p Fiji package software (https://imagej.net/Fiji). Equal protein concentrations from cell lysates were charged on gel prior to quantification. STING expression was used for normalization. The results are presented in the form of histograms and error bars represent the standard error mean (SEM) for n = 3 independent experiments. Statistical significance was assessed by t-test; *p < 0.05, **p < 0.01, ***p < 0.001 and ****p < 0.0001 (threshold of significance of 5%).

#### Image quantification

Fluorescence microscopy images were analyzed and quantified using QuPath-0.4.3 software (https://qupath.github.io/). The analysis of NiV N antigen on brain organs of NiV-infected mice was performed through positive pixel detection. The analysis of mean intensity/cell of phospho-STING and Mitotracker on HPMEC cells was performed on n = 50 events on average for each of 4 independent replicates. The measurement of nuclear mean intensity of NF-κB p65 fraction on HPMEC cells was performed on at least n = 130 events per condition. Statistical significance was assessed by a one-way ANOVA, followed by Tukey’s or Dunnett’s multiple comparisons test; *p < 0.05, **p < 0.01, ***p < 0.001 and ****p < 0.0001 (threshold of significance of 5%).

Fluorescence spectra of cGAS and IFI16 in HeLa cells, representing fluorescence intensity distribution as a function of cellular area, were obtained with ImageJ software and graphically represented using GraphPad Prism.

## Supporting information

S1 Fig STING-associated sensors cGAS and IFI16 are involved in the control of MeV infection. WT, STING KO, cGAS KO or IFI16 KO THP-1 cells were infected with NiV-eGFP at a MOI of 0.3 (A) or MeV-eGFP at a MOI of 0.1 (B) for 48h. **(A-B)** eGFP expression was evaluated by fluorescence microscopy in NiV-eGFP (A) or MeV-eGFP (B) infected cells.
(TIF)

S2 Fig STING is activated following syncytia formation induced by NiV and MeV infection. **(A-B)** Band intensity from western blots presented in Fig 2B and 2D were calculated by densitometry using ImageJ on results from 3 independent replicates. All samples were analyzed using t-test, ns (not significant); *p<0.05; **p<0.01. **(C)** Expression of NiV-F and NiV-G was measured by flow cytometry in 293T and 293 cells prior to co-culture. **(D)** 293 and 293T cells were tested for IFI16, cGAS and STING expression by western blot analysis.
(TIF)

S3 Fig Evaluation of NiV-induced syncytia formation on mitochondrial and DNA damage. **(A)** HeLa cells WT or stably expressing NiV-F or NiV-G were cultured individually or co-cultured, treated or non-treated with VIKI fusion inhibitor peptide at 1 μM and incubated for 48h. The scheme was created with Biorender (agreement number: CR26WU6KQ4). **(B)** Expression of NiV-F and NiV-G in HeLa cells was measured by flow cytometry. (C-F) HeLa cells WT or stably expressing NiV-F or NiV-G were cultivated individually or co-cultured, treated or non-treated with VIKI fusion inhibitor peptide at 1 μM and incubated for 48h (n = 3). Cells were stained with Mitotracker Orange at 100 nM, fixed, stained with anti-cGAS (C) or anti-IFI16 (E) antibodies and analyzed by confocal microscopy. The indicated region of the slide is enlarged (white rectangle), fluorescent spectrum of the cGAS (D) and IFI16 (F) staining in the cell nucleus and/or cytoplasm was determined by ImageJ software and graphical presentation of the expression profile was obtained using GraphPad Prism.
(TIF)

S4 Fig Mitochondrial DNA is responsible for STING activation through its sensing by cGAS and IFI16 during MeV and NiV infection. **(A)** HeLa cells were infected with NiV-eGFP or MeV-eGFP at a MOI of 0.3 and treated or non-treated with VIKI or HRC4 fusion inhibitor peptides, respectively, at 1 μM 6h post infection. 48h later, the expression of NiV-M or MeV-N proteins was analyzed by western blot. **(B)** HeLa cells were infected with MeV-eGFP at a MOI of 0.3 and treated or non-treated with HRC4 fusion inhibitor peptides at 1 μM 6h post infection. 48h later, the expression of cGAS, IFI16 and GAPDH proteins in nuclear and cytoplasmic fractions was analyzed by western blot. **(C)** Total DNA was extracted from non-infected HeLa cells and presence of mitochondrial DNA (mt 1 and mt 2) and nuclear DNA (Nuc DNA 1 and Nuc DNA 2) was assessed by qPCR (n = 3). **(D)** Whole cell, cytoplasmic extracts (CE) or nuclear extracts (NE) fractions were obtained from non-infected HeLa cells. Immunoprecipitation was performed on the three fractions with anti-Tri-Methyl-Histone H3 (3m-H3) and IgG antibodies. 3m-H3 immunoprecipitation was verified by western blot. Purified DNA from immunoprecipitated samples was analyzed by qPCR for nuclear DNA (Nuc DNA 1) and mitochondrial DNA (mt 1) targets. Results are represented as fold change of target DNA bound to 3m-H3 compared to IgG control. **(E-H)** HeLa cells were infected with NiV-eGFP (C, E) or MeV-eGFP (D, F) at a MOI of 0.3 and treated or non-treated with VIKI or HRC4 fusion inhibitor peptides, respectively, at 1 μM 6h post infection. 48h post infection, cytoplasm was extracted and cGAS and IFI16 proteins were immunoprecipitated from cytoplasmic extract. Purified DNA from immunoprecipitated cGAS (C, D) or IFI16 (E, F) was analyzed by qPCR for two nuclear (Nuc DNA 1 and Nuc DNA 2) DNA regions.
(TIF)

S5 Fig Characterization of mitochondrial and nuclear qPCR targets. Two mitochondrial regions (A, B) and two nuclear genes (C, D) targeted by qPCR during co-immunoprecipitation analysis were sequenced in order to confirm their mitochondrial or nuclear genome localization. Sanger sequences were manually aligned to the following reference sequences obtained from Genbank: Mt1 and Mt2: mitochondrion genome OR088596, Nuc1: laminin subunit alpha 4 NM_002290.5 and Nuc2: Janus kinase 1 NM_001321853.1.
(TIF)

S1 Data All raw data are available and have been provided as Supporting Information. (XLSX)
