# Supplementary figures and images for: Multifaceted activation of STING axis upon Nipah and measles virus-induced syncytia formation
Source: PLoS Pathog. 2024 Sep 16;20(9):e1012569. doi: 10.1371/journal.ppat.1012569 (PMC11426520; doi:10.1371/journal.ppat.1012569)

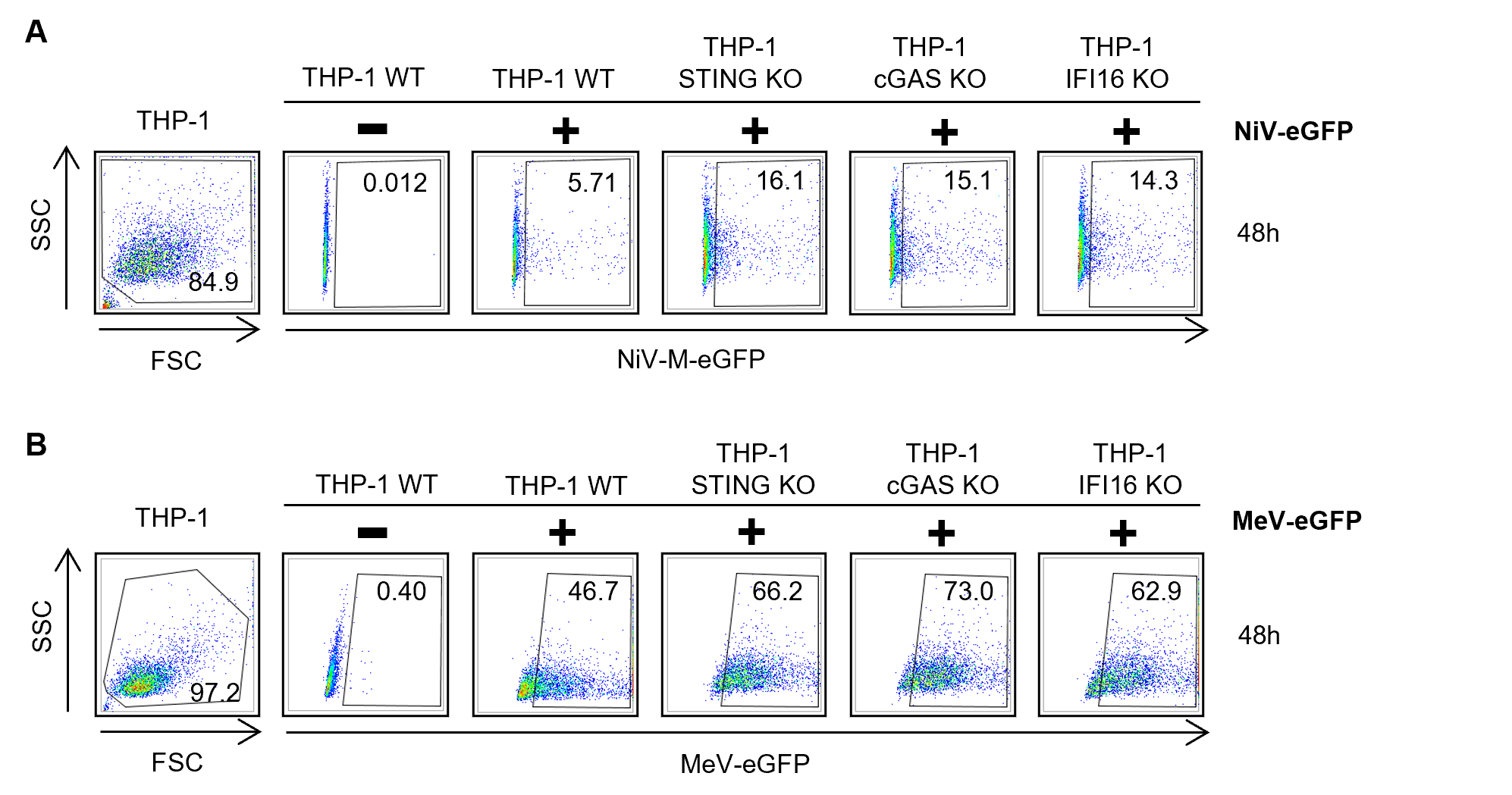

Supplement: S1 Fig — WT, STING KO, cGAS KO or IFI16 KO THP-1 cells were infected with NiV-eGFP at a MOI of 0.3 (A) or MeV-eGFP at a MOI of 0.1 (B) for 48h. (A-B) eGFP expression was evaluated by fluorescence microscopy in NiV-eGFP (A) or MeV-eGFP (B) infected cells. (TIF) [file ppat.1012569.s001.tif]

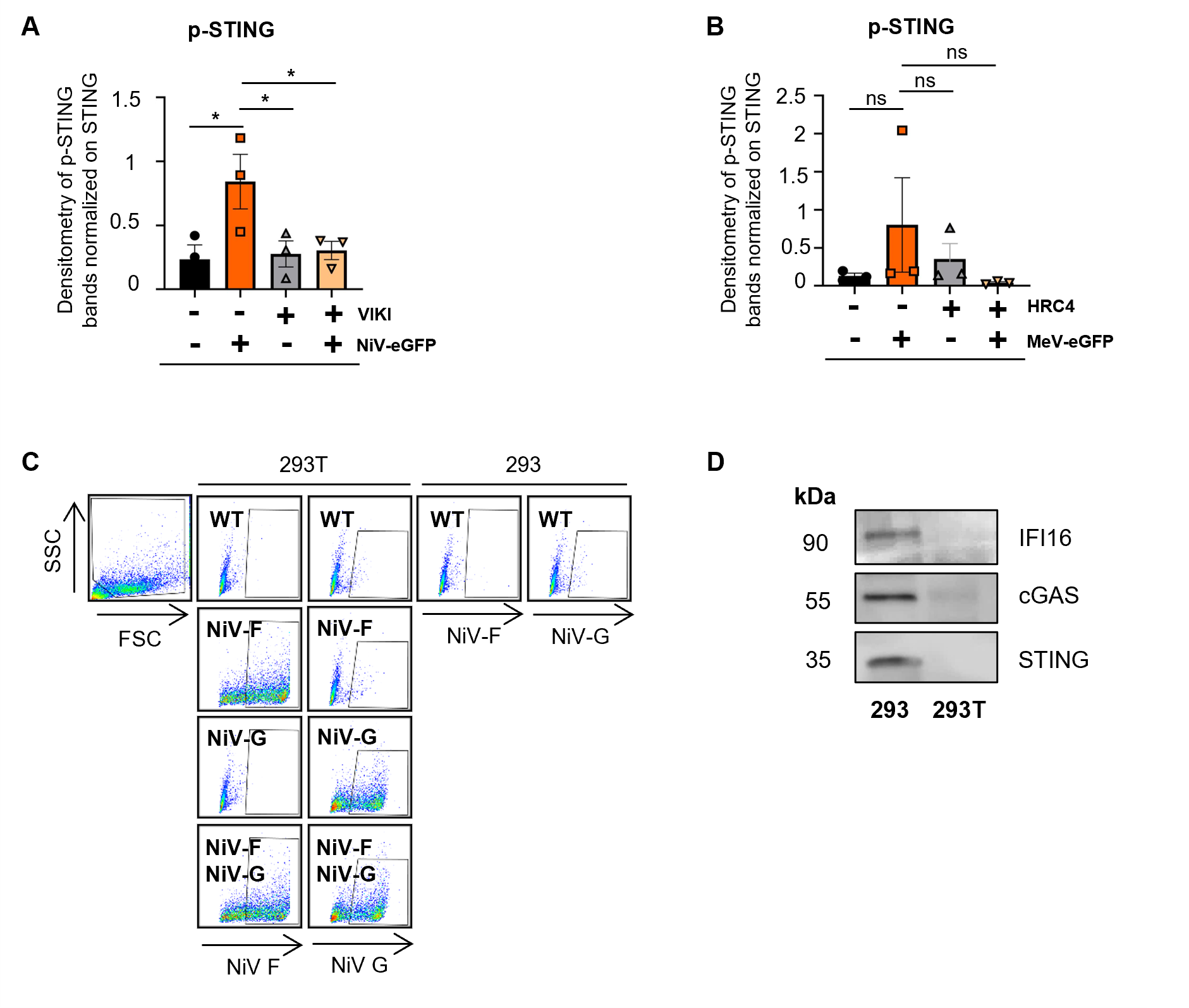

Supplement: S2 Fig — (A-B) Band intensity from western blots presented in Fig 2B and 2D were calculated by densitometry using ImageJ on results from 3 independent replicates. All samples were analyzed using t-test, ns (not significant); *p<0.05; **p<0.01. (C) Expression of NiV-F and NiV-G was measured by flow cytometry in 293T and 293 cells prior to co-culture. (D) 293 and 293T cells were tested for IFI16, cGAS and STING expression by western blot analysis. (TIF) [file ppat.1012569.s002.tif]

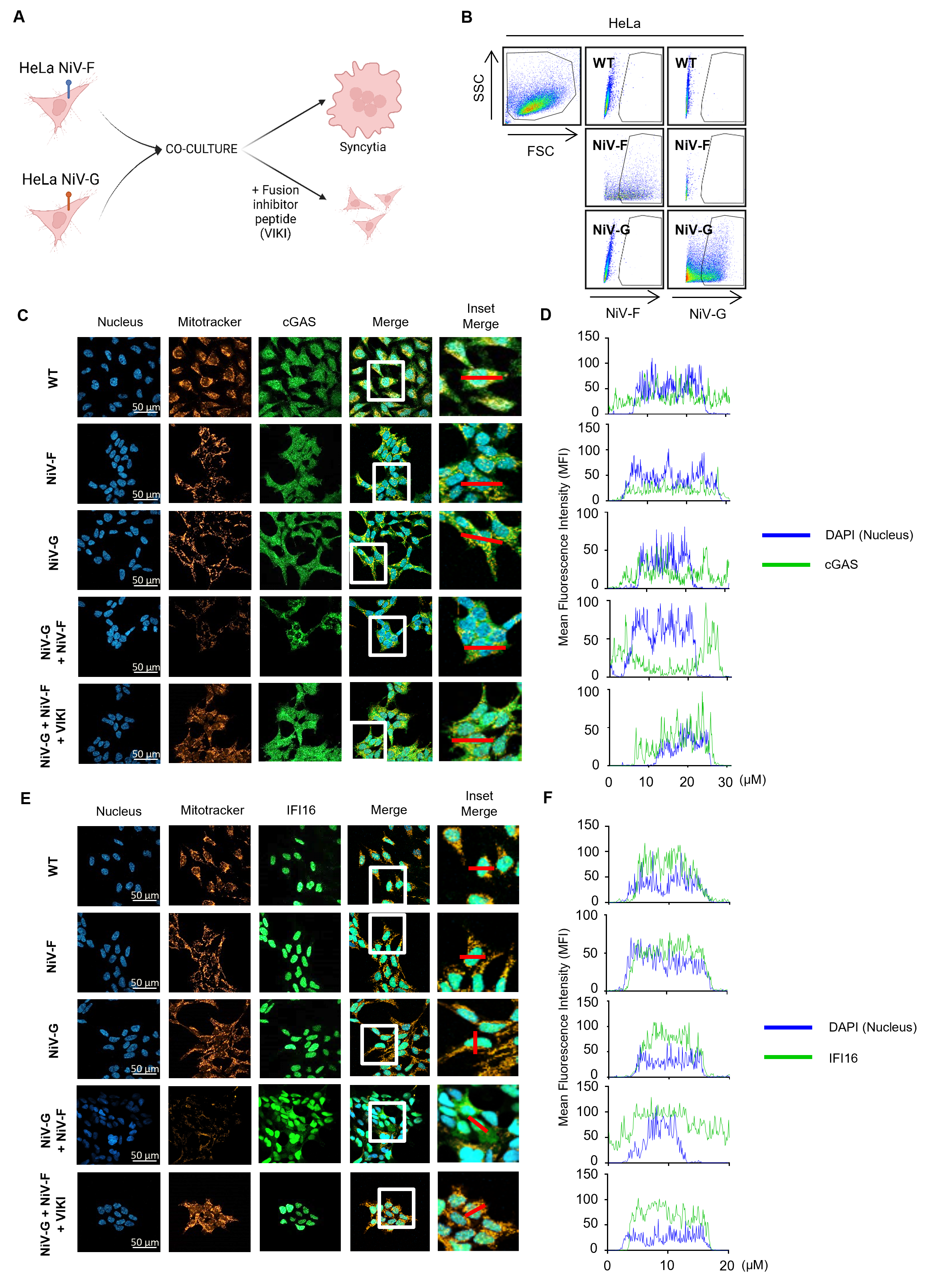

Supplement: S3 Fig — (A) HeLa cells WT or stably expressing NiV-F or NiV-G were cultured individually or co-cultured, treated or non-treated with VIKI fusion inhibitor peptide at 1 μM and incubated for 48h. The scheme was created with Biorender (agreement number: CR26WU6KQ4). (B) Expression of NiV-F and NiV-G in HeLa cells was measured by flow cytometry. (C-F) HeLa cells WT or stably expressing NiV-F or NiV-G were cultivated individually or co-cultured, treated or non-treated with VIKI fusion inhibitor peptide at 1 μM and incubated for 48h (n = 3). Cells were stained with Mitotracker Orange at 100 nM, fixed, stained with anti-cGAS (C) or anti-IFI16 (E) antibodies and analyzed by confocal microscopy. The indicated region of the slide is enlarged (white rectangle), fluorescent spectrum of the cGAS (D) and IFI16 (F) staining in the cell nucleus and/or cytoplasm was determined by ImageJ software and graphical presentation of the expression profile was obtained using GraphPad Prism. (TIF) [file ppat.1012569.s003.tif]

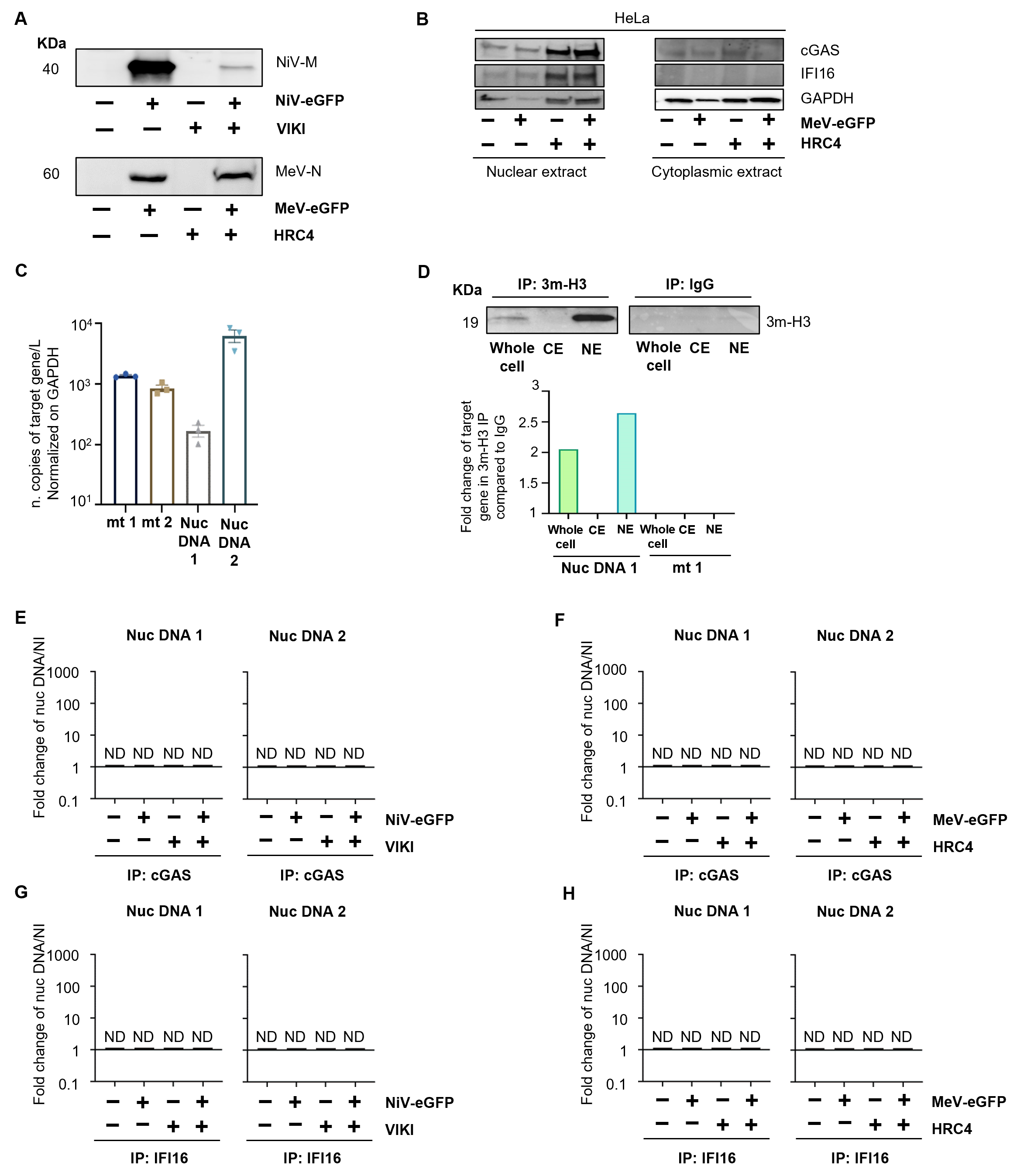

Supplement: S4 Fig — (A) HeLa cells were infected with NiV-eGFP or MeV-eGFP at a MOI of 0.3 and treated or non-treated with VIKI or HRC4 fusion inhibitor peptides, respectively, at 1 μM 6h post infection. 48h later, the expression of NiV-M or MeV-N proteins was analyzed by western blot. (B) HeLa cells were infected with MeV-eGFP at a MOI of 0.3 and treated or non-treated with HRC4 fusion inhibitor peptides at 1 μM 6h post infection. 48h later, the expression of cGAS, IFI16 and GAPDH proteins in nuclear and cytoplasmic fractions was analyzed by western blot. (C) Total DNA was extracted from non-infected HeLa cells and presence of mitochondrial DNA (mt 1 and mt 2) and nuclear DNA (Nuc DNA 1 and Nuc DNA 2) was assessed by qPCR (n = 3). (D) Whole cell, cytoplasmic extracts (CE) or nuclear extracts (NE) fractions were obtained from non-infected HeLa cells. Immunoprecipitation was performed on the three fractions with anti-Tri-Methyl-Histone H3 (3m-H3) and IgG antibodies. 3m-H3 immunoprecipitation was verified by western blot. Purified DNA from immunoprecipitated samples was analyzed by qPCR for nuclear DNA (Nuc DNA 1) and mitochondrial DNA (mt 1) targets. Results are represented as fold change of target DNA bound to 3m-H3 compared to IgG control. (E-H) HeLa cells were infected with NiV-eGFP (C, E) or MeV-eGFP (D, F) at a MOI of 0.3 and treated or non-treated with VIKI or HRC4 fusion inhibitor peptides, respectively, at 1 μM 6h post infection. 48h post infection, cytoplasm was extracted and cGAS and IFI16 proteins were immunoprecipitated from cytoplasmic extract. Purified DNA from immunoprecipitated cGAS (C, D) or IFI16 (E, F) was analyzed by qPCR for two nuclear (Nuc DNA 1 and Nuc DNA 2) DNA regions. (TIF) [file ppat.1012569.s004.tif]

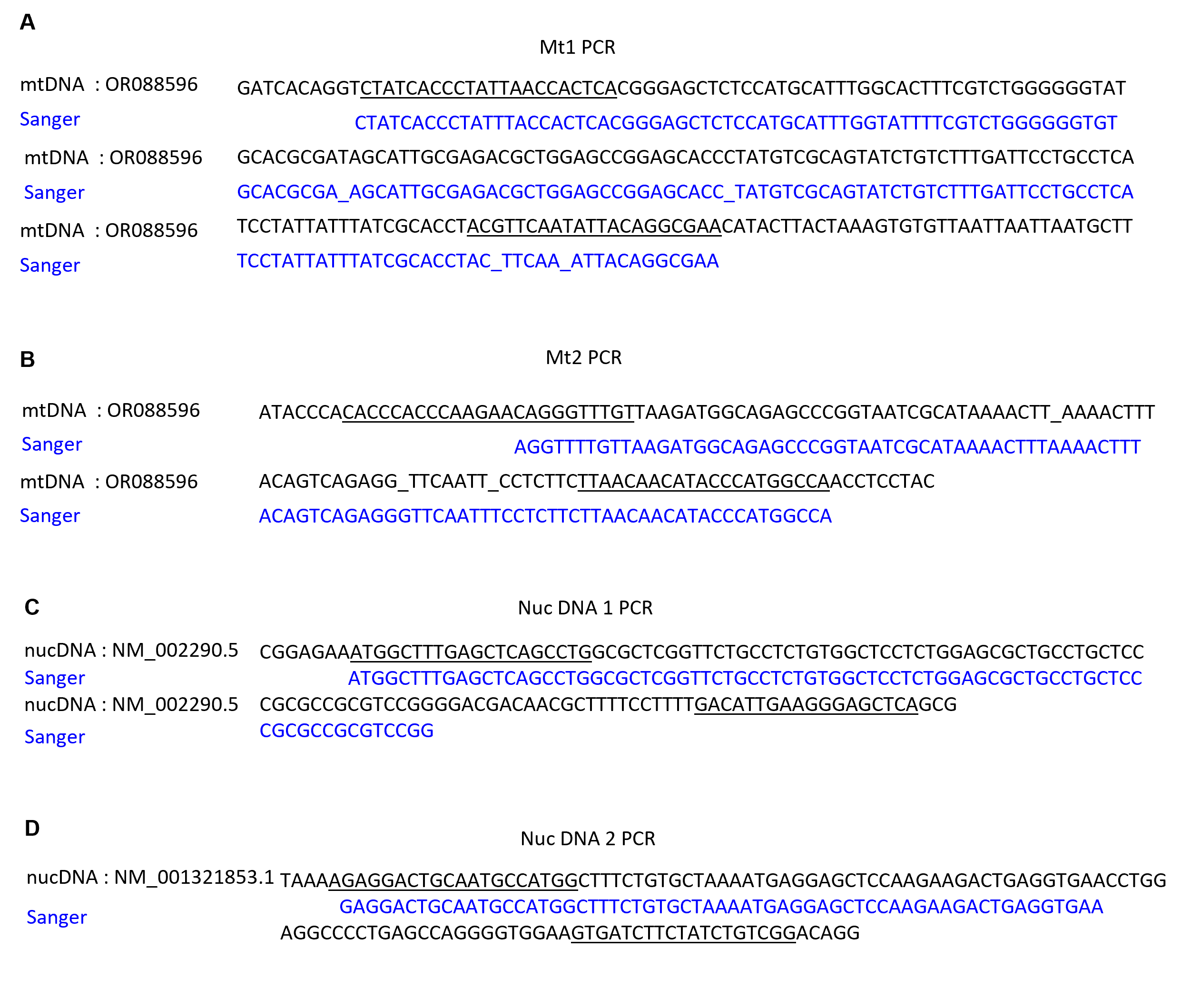

Supplement: S5 Fig — Two mitochondrial regions (A, B) and two nuclear genes (C, D) targeted by qPCR during co-immunoprecipitation analysis were sequenced in order to confirm their mitochondrial or nuclear genome localization. Sanger sequences were manually aligned to the following reference sequences obtained from Genbank: Mt1 and Mt2: mitochondrion genome OR088596, Nuc1: laminin subunit alpha 4 NM_002290.5 and Nuc2: Janus kinase 1 NM_001321853.1. (TIF) [file ppat.1012569.s005.tif]
